# A Global Identification of Protein Disulfide Isomerases from ‘duli’ Pear (*Pyrus betulaefolia*) and Their Expression Profiles under Salt Stress

**DOI:** 10.3390/genes15080968

**Published:** 2024-07-23

**Authors:** Hao Zhang, Yuyue Zhang, Kexin Cui, Chang Liu, Mengya Chen, Yufan Fu, Zhenjie Li, Hui Ma, Haixia Zhang, Baoxiu Qi, Jianfeng Xu

**Affiliations:** 1 College of Horticulture, Hebei Agricultural University, Baoding 071001, China; m18233088367@163.com (H.Z.); 15614841007@163.com (Y.Z.); 19801564669@163.com (K.C.); 18033516510@163.com (C.L.); cmy001027@163.com (M.C.); 18131131319@163.com (Y.F.); 18631681891@163.com (Z.L.); yymh@hebau.edu.cn (H.M.); zhx2323a@163.com (H.Z.); 2 Research Center for Pear Engineering and Technology of Hebei Province, Baoding 071001, China; 3 School of Pharmacy and Biomolecular Sciences, Liverpool John Moores University, James Parsons Building, Byrom Street, Liverpool L3 3AF, UK

**Keywords:** ‘duli’ pear, *Pyrus betulaefolia*, protein disulfide isomerase, salt stress

## Abstract

Protein disulfide isomerases (PDIs) and PDI-like proteins catalyze the oxidation and reduction in protein disulfide bonds, inhibit aggregation of misfolded proteins, and participate in isomerization and abiotic stress responses. The wild type ‘duli’ pear (*Pyrus betulaefolia*) is an important rootstock commonly used for commercial pear tree grafting in northern China. In this study, we identified 24 *PDI* genes, named *PbPDIs*, from the genome of ‘duli’ pear. With 12 homologous gene pairs, these 24 *PbPDIs* distribute on 12 of its 17 chromosomes. Phylogenetic analysis placed the 24 PbPDIs into four clades and eleven groups. Collinearity analysis of the *PDIs* between *P. betulaefolia*, *Arabidopsis thaliana*, and *Oryza sativa* revealed that the *PbPDIs* of ‘duli’ pear show a strong collinear relationship with those from Arabidopsis, a dicot; but a weak collinear relationship with those from rice, a monocot. Quantitative RT-PCR analysis showed that most of the *PbPDIs* were upregulated by salt stress. Identification and expression analysis of ‘duli’ pear *PbPDIs* under salt stress conditions could provide useful information for further research in order to generate salt-resistant rootstock for pear grafting in the future.

## 1. Introduction

Protein disulfide isomerases (PDI), members of the thioredoxin (TRX) superfamily, are thiol-disulfide oxidoreductase chaperones. They reside in the endoplasmic reticulum (ER) as well as in the nucleus, cytoplasm, and extracellular space, where they catalyze the synthesis of proteins or the formation, reduction, and rearrangement of disulfide bonds in the target proteins [1,2,3]. PDIs play a key role in severing disulfide bonds to form protein conformations with the lowest free energy, thereby aiding newly synthesized proteins to reform disulfide bonds and produce correctly folded structures [4,5]. Concurrently, PDIs can also bind to misfolded or denatured proteins, preventing the accumulation of aggregates that affect normal cell metabolism [6,7]. Therefore, PDIs are particularly important for the correct folding and stability of proteins.

PDI and PDI-like (PDIL) proteins contain at least one redox-active TRX domain that is responsible for the alteration of disulfide bonds. Classical human PDIs typically consist of four TRX-like domains: a, a’, b, and b’; one linker (X); and one C-terminal extension domain (C) [8]. The a and a’ domains share high homology with TRX, have a secondary structure consisting of dense α helices and β chains (β-α-β-α-β-β-β-α), and contain an active Cys-Gly-His-Cys (CGHC) motif that is essential for the redox and isomerization of peptides [9,10], whilst the b and b’ domains do not share sequence homology with TRX and lack the -CXXC- motif. Although the b and b’ domains have a secondary structure and substrate binding ability similar to the active TRX domain, they do not have specific activities of redox and isomerization [11]. The C domain is rich in acidic residues and is typical for Ca^2+^ binding proteins [12]. This domain usually ends with KDEL/GKNF/VASS, which are essential for ER retention [13].

Genes encoding for PDIs have been identified in a variety of plant species, including 22 in *A. thaliana* [14], 32 in *Brassica rapa* [15], 19 in *Solanum lycopersicum* L [16], 17 in *Medicago truncatula* [17], 12 in *Bryza sativa*, *Brachpodium distachyon* and *Zea mays*, respectively, 10 in *Vitis vinifera*, 9 in *Triticum aestivum*, and 8 in *Cicer arietinum* [18]. In general, PDI proteins are divided into 11 groups. Group I–V PDIs usually contain two, whereas group VI–XI only have one TRX active domain [15].

PDIs have diverse functions and are widely involved in the regulation of plant growth, development, and stress response. In *A. thaliana*, *PDI1* is upregulated under different abiotic stresses; its overexpression increased seed germination rate and promoted root growth under different abiotic stresses, emphasizing the role of PDIs in abiotic stress tolerance, which is directly related to its disulfide isomerase activity [19,20]. *PDI9* functions in exine formation and heat tolerance of pollen [19,20]. *PDI6* regulates photoinhibition in a photoregulatory manner [21]. Mutation in *PDIL2-1* causes delays in embryo sac maturation, which affects pollen tube orientation and attraction [22]. In wheat, *TaPDI1* is directly related to gluten quality. Expression of *TaPDI* is positively correlated to the processing characteristics of wheat dough by maintaining the GMP accumulation level [23,24]. The transcript levels of *TaPDIs* are significantly increased during starch endosperm filling. PDIs also play important roles in the formation of disulfide bonds in seed storage proteins [25,26]. Most *PDI* genes in *S. lycopersicum* L are highly induced by heat, salt, and abscisic acid (ABA), while a few *PDI* genes are also induced by cold, nutrient, and nutrient/water deficit stress. In *B. rapa*, the transcripts of majority of the *PDIs* are elevated by salt and drought stress, whereas the rest are by low temperature and ABA stress [16]. PDIs are also involved in pathogen resistance. For example, 14 of the 32 *BrPDIs* in *B. rapa* were significantly upregulated after infection with *Fusarium oxysporum f.sp.,* while overexpression of *PDI-V* in susceptible wheat varieties confers high resistance to powdery mildew [15]. Resistance to tomato yellow leaf curl virus by SlPDI can be achieved by enhancing the protein-folding function of the ER as well as by promoting the synthesis and conformation of antioxidant-related proteins [27].

Salt stress is an important obstacle that limits the economic cultivation of fruit trees, including pear [28]. As a cash crop, pear plays an important role in poverty alleviation and rural revitalization in China. Although the WT ‘duli’ pear fruits are not edible, it is widely used as a common rootstock for grafting to commercial pear trees in Northern China due to its strong growth and resistance to salt-alkali, drought, cold, and barren stress. Importantly, it has high affinity with elite pear cultivars compared to the more traditional European quince rootstock. Given the role of PDIs in abiotic stress tolerance in several plant species, we sought to survey the recently sequenced genome of ‘duli’ pear, aiming to identify *PDI* homologous genes and explore their biological functions. This may provide vital information for future research on the PDIs in ‘duli’ in order to obtain salt-tolerant rootstock via genetic improvement.

## 2. Materials and Methods

### 2.1. Identification and Sequence Analysis of PbPDIs

The genome file of ‘duli’ pear (*P. betulaefolia* Genome v1.0) was obtained from the Genome Database for Rosaceae (https://www.rosaceae.org/, accessed on 30 September 2023). The Arabidopsis PDI protein sequences were obtained using ‘PDI’ as the keyword to search The Arabidopsis Information Resource (https://www.arabidopsis.org/, accessed on 30 September 2023) [29]. These sequences are used as references to blast the genome of ‘duli’ pear. The resulting putative ‘duli’ PDI and the Arabidopsis PDIs were inputted in the National Center for Biotechnology Information Conserved Domains Database (NCBICDD) to screen for those protein sequences containing the TRX domain (PF00085) [30]. All the protein sequences containing the TRX domain were downloaded from the Pfam database (http://pfam.xfam.org/, accessed on 30 September 2023), and homology comparison with all ‘duli’ protein sequences was performed. This resulted in the identification of 24 such protein sequences that were proposed to be members of the PDI family of ‘duli’ pear, named hereafter as PbPDIs.

We next catalogued the gene entry number, locus name, and coding sequence of all the 24 *PbPDIs*. TBtools software v2.096 (a Toolkit for Biologists integrating various biological data-handling tools) was used to analyze and visualize the intron/exon construction of *PbPDI* genes. The promoter region of 2000 bp upstream of the start codon (ATG) of each gene was analyzed using the PlantCare database to predict the presence of cis-acting elements (https://bioinformatics.psb.ugent.be/webtools/plantcare/html/, accessed on 2 October 2023) [31]. The protein molecular weight (Mw), isoelectric point (PI), instability factor, coefficient of fat, and hydrophilic information were predicted via ExPASy (https://web.expasy.org/protparam/, accessed on 2 October 2023) [32]. The subcellular localizations of PbPDIs were predicted with WoLF Protein Subcellular Localization Prediction Tool (PSORT) (https://www.genscript.com/wolf-psort.html/, accessed on 2 October 2023).

### 2.2. Phylogenetic and Conserved Motif Analysis of the PbPDIs

The full-length PbPDI protein sequences of ‘duli’ pear (*P. betulaefolia*) and those of *A. thaliana*, *Z. mays,* and *B. rapa* [33] were compared using the Molecular Evolutionary Genetics Analysis version 11 (MEGA11) software. The neighbor-joining (NJ) algorithm was used for phylogenetic analysis, with a bootstrap value of 1000.

To test the diversity of functional protein motifs in PbPDI proteins, we used the Multiple Em for Motif Elicitation (MEME) online website (http://meme-suite.org/, accessed on 4 October 2023) to identify conserved protein motifs using parameters of 10 for the maximum motif number, and the motif length between 6 and 50 amino acids [34].

### 2.3. Chromosome Localization, Gene Duplication, and Collinearity Analysis

The locations of the *PbPDI* genes and their subgenomic information were obtained from the ‘duli’ genome file and visualized using TBtools v2.096 [35]. According to the criteria proposed by Kong et al. [36], we used TBtools to identify gene duplications between the *PbPDIs*. Genes were considered duplicates when their identity and query coverage were >80%. Tandem repeats were labeled as arrays of two or more homologous genes within 100 kb, otherwise they were labeled as fragment repeats. The Nei–Gojobori method (1986) and MEGA 11.0 software were used to determine the synonymous (Ks) and nonsynonymous (Ka) nucleotide substitution rates of the duplicated *PbPDI* gene pairs [37]. The selection pattern was determined based on the Ka/Ks ratio, where >1, <1, and =1 were regarded as positive, purified, and neutral selections, respectively. The differentiation time (T) for each duplicate gene pair is calculated using the formula: T = Ks/2r MYA (millions of years ago), where Ks is the synonym substitution rate per site and r is the substitution rate of 1.5 × 10^−8^ per site per year in dicotyledonous plants [38].

Interspecies collinearity analysis was carried out between *P. betulaefolia* and *A. thaliana*, as well as between *P. betulaefolia* pear and *O. sativa* L. *PDIs*, using TBtools software [35].

### 2.4. Plant Materials, RNA Extraction, cDNA Synthesis, and Real-Time PCR

Tissue-cultured seedlings of ‘duli’ pear (provided by the Pear Technology Innovation Center of Hebei Province, China) were cultured at 25 °C under a 16 h/8 h (light/dark) cycle on Murashige and Skoog medium (MS, pH 5.8) containing sucrose (30 g/L), 6-benzylaminopurine (6-BA, 1.0 mg/L), indole-3-butyric acid (IAA, 0.1 mg/L), and agar (7 g/L). The seedlings were subcultured under the same conditions every 20–30 days. For salt treatment, tissue-cultured seedlings were placed on MS medium containing NaCl (150 mM). Samples (3 seedlings at each time point) were collected at 6, 12, and 24 h. Untreated samples (0 h) were used as control. All samples were frozen in liquid nitrogen immediately and stored at −80 °C for RNA extraction.

The Plant RNA Kit (OMEGA bio-tek, Canton, China) was used to extract total RNA according to the manufacturer’s instructions. The RNA concentration was estimated using a Mettler Toledo UV5 nano UV–VIS spectrophotometer (Mettle Toledo, Zurich, Germany). The Prime Script TMRT Reagent Kit with gDNA Eraser (Tiangen, Beijing, China) was used to synthesize the first strand cDNA using 1 µg total RNA for each reaction.

For real-time PCR, gene-specific primers were designed and analyzed for specificity by melting curve analysis (Supplementary Table S1). *PbACT* (LOC103926846) was used as the reference gene for normalization. Using a FastReal qPCR PreMix kit (Tiangen, Beijing, China), the PCR reaction (20 µL) was set up to contain 10 µL 2× FastReal qPCR PreMix, 100 ng cDNA, 0.6 µL forward/reverse primers (10 µM), 0.4 µL 50× ROX, and ultra-pure water. Fluorescence qPCR was performed in an optical 96-well plate using a Thermo Field QuantStudio 5 qPCR instrument (Thermo Fisher Scientific, Waltham, MA, USA) with the following conditions: 95 °C predenaturation for 2 min, and 40 cycles of denaturation at 95 °C for 5 s, annealing at 60 °C for 10 s, and extension at 72 °C for 15 s. The relative expression level of each gene was analyzed using the 2^−∆∆Ct^ method [39], using the value of 0 h sample as 1. The obtained data were analyzed using Data Processing System software (DPS 9.01) (http://www.dpsw.cn/, accessed on 11 November 2023) to determine the significance of the relative representative data.

## 3. Results

### 3.1. 24 PDIs Were Identified in the Genome of ‘duli’ Pear

**Figure 1:**
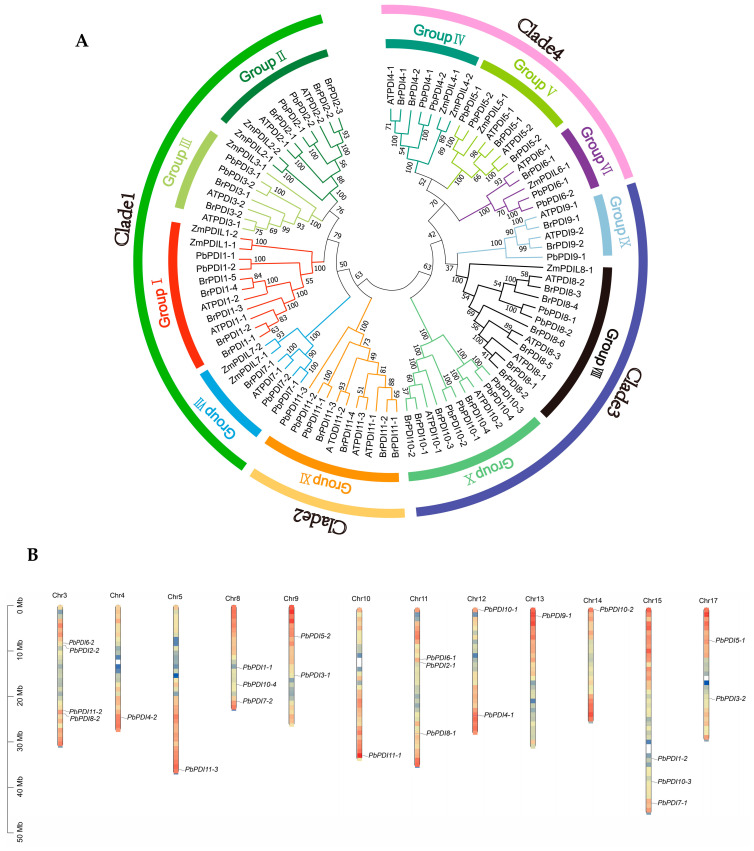

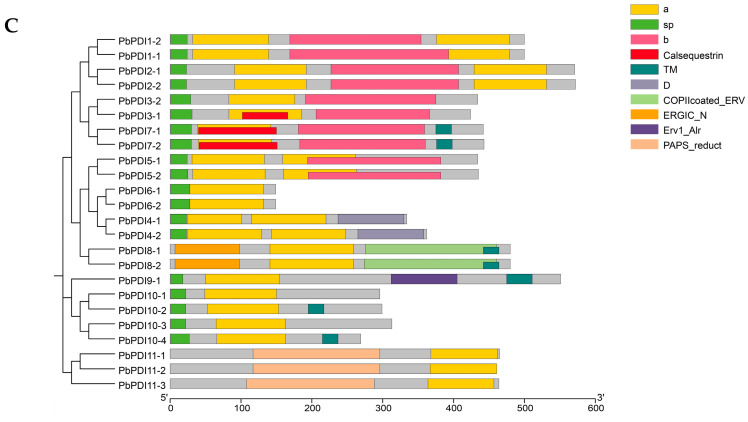
Identification of 24 PbPDIs from the genome of ‘duli’ pear (*P. betulaefolia*). (**A**) Phylogenetic relationships between the 24 PbPDIs of *P. betulaefolia*, *A. thaliana*, *B. rapa*, and *Z. mays*. MEGA11 software was used to construct the phylogenetic tree with 1000 bootstrap repeats using the NJ algorithm. Proteins are divided into 11 groups (I–XI), belonging to four clades. The amino acid sequences used in the phylogenetic analysis and their entry numbers are listed in Supplementary Table S1. (**B**) Distribution of the 24 *PbPDIs* on 12 of the 17 chromosomes. Chromosome numbers are indicated at the top of each chromosome. Chromosome size and gene location were estimated using the megabase pair (Mbp) scale indicated on the left. (**C**) Prediction of the structural domains of the 24 PbPDIs. Different domains are color-coded as indicated on the right. SP, signal peptide (green); a (yellow) and b (pink), TRX-like domains; Calsequestrin (red), N-terminal calcium binding domain calsequestrin; D domain (lilac), Erp29c; ERGIC_N domain (orange), ER-Golgi Intermediate Compartment_N domain; C_ERV (COPII-coated ERV) domain (aqua); Evr1_Air domain (modena); PAPS_reduct domain (light pink), phosphoadenosine phosphosulfate reductase domain; TM (dark green), transmembrane domain.

We identified 24 PDIs from ‘duli’ pear genome and designated them as PbPDI1-1-PbPDI11-3 after homology comparison of the protein sequences of *P. betulaefolia* with the 21 AtPDIs of *A. thaliana*, 12 ZmPDIs of *Z. mays*, and 32 BrPDIs of *B. rapa* (Figure 1A, see also the next section below). They are distributed on 12 of the 17 chromosomes, where chromosome 3 has the highest number of 4, followed by 3 on chromosome 8, 11, and 15, respectively, while chromosomes 1, 2, 6, 7, and 16 do not have any *PbPDIs* (Figure 1B). They are 2551-21,142 bp long with ORFs ranging from 450 bp (*PbPDI6-1* and *PbPDI6-2*) to 1719 bp (*PbPDI2-2*), and the number of exons from 4 to 15. The predicted lengths of their encoded proteins range from 149 (PbPDI6-1 and PbPDI6-2) to 571 (PbPDI2-2) amino acids with molecular weight (MW) between 16.6 (PbPDI6-1) and 63.9 (PbPDI2-2) kDa. The isoelectric points (PI) of the proteins are between 4.6 (PbPDI2-2) and 9.8 (PbPDI10-1) (Table 1). They are predicted to localize at the plasma membrane (PM, 7 PbPDIs), endoplasmic reticulum (ER, 4 PbPDIs), the extracellular space (4 PbPDIs), the vacuole membrane (2 PbPDIs), cytoplasm (1 PbPDI), and in chloroplast (6 PbPDIs) (Table 1).

### 3.2. Phylogeny and Protein Structure Analysis

The phylogenetic tree constructed with the 24 PbPDIs of ‘duli’ and other PDIs from *A. thaliana*, *Z. mays,* and *B. rapa* revealed that these 89 PDIs can be divided into four clades which are further divided into 11 groups (Figure 1A). Clade 1 contains the largest number (33) of members, including groups I, II, III, and VII, of which members of groups I and II have an active TRX domain at the N- and C-terminii, and members of groups III and VII have a single active TRX domain at their N-terminus (green line, Figure 1A). Clade 2 includes only group XI; their members all contain a 3′-phospho-adenosine-5′-phospho-sulfate (PAPS) reductase domain and an active TRX domain at the C-terminus (light orange, Figure 1A). Clade 3 includes groups IV, V, and VI, of which proteins in groups IV and V contain two active TRX domains in tandem at their N-terminus, whereas group VI contains a single N-terminal active TRX domain (dark blue, Figure 1A). Clade 4 consists of groups VIII, IX, and X, where all the members contain an active TRX domain (pink, Figure 1A).

Further analysis of the 24 PbPDI proteins of ‘duli’ pear show that except the ones in group III, VIII, and X, all others have at least one active TRX domain, which contains the isomerase and redox activity -CXXC- motif. Members of Group VIII and X have noncharacteristic active sites of CYWS and CPFS, respectively (Table 2). Of the 24 PbPDI proteins, 19 have predicted N-terminal signal peptides (SP) required for polypeptide translocation, seven have specific transmembrane domains, and six have C-terminal KDEL/GKNF/VASS signals for ER retention, which is consistent with their subcellular localization prediction (Table 1 and Table 2, Figure 1C).

**Figure 2:**
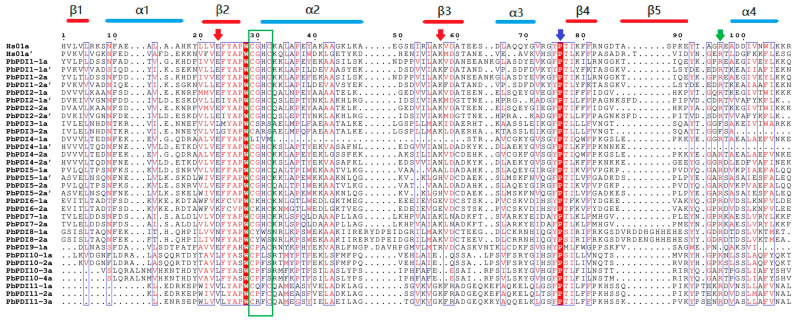
Multiple sequence alignment between the a-type domain of PbPDI protein and the a-type domain of classical human PDI. The TRX-like domain in the PbPDIs was annotated using the HMM network tool (http://pfam.xfam.org/, accessed on 30 September 2023) and compared using Clustal Omega (http://www.clustal.org/omega/, accessed on 2 October 2023). The composition of the secondary structure is represented by blue (α-helices) and red (β-sheets) lines. The red arrow indicates the Glu–Lys charged pair located near the active site, the green arrow indicates the conserved Arg (R) between β5 and α4 of each catalytic domain, and the blue arrow indicates the vicinity of each active site. The -CXXC- motif is marked with a green box. Identical amino acids are marked as red letters and boxed in blue boxes while the amino acids W and P are shaded red.

We compared the a(a’)-type domain of the PbPDI proteins with the a(a’) domain of the classical human PDIs and found that the TRX-like domain contains a secondary structure consisting of dense α helices, β chains (β-α-β-β-β-β-α), and the -CXXC- motif (green box, Figure 2). Most of the PbPDIs have conserved arginine (green arrow), glutamate (red arrow), proline (blue arrow), and lysine residues (red arrow) (Figure 2). Notably, the a-type domain of the PDI4-1 protein lacks the -CXXC- motif and conserved lysine, and the a’-type domain lacks conserved arginine.

### 3.3. Exon and Intron Distribution and Conserved Motif Analysis

**Figure 3:**
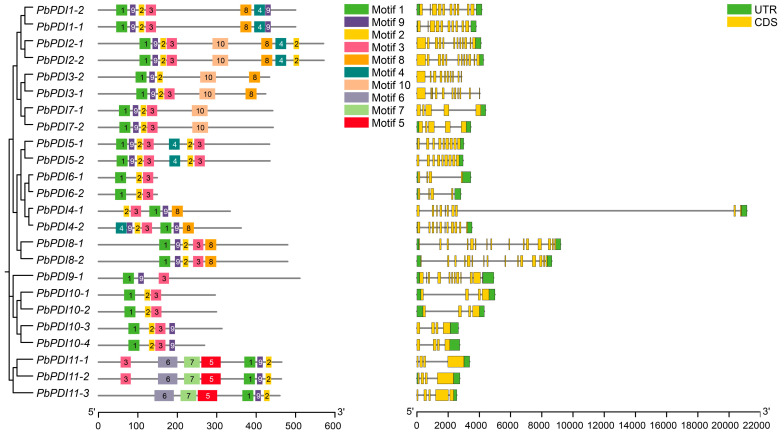
The ten conserved motifs and gene structure of the 24 PbPDIs of ‘duli’ pear (*P. betulaefolia*). The conserved motifs were analyzed using MEME software (http://meme-suite.org/, accessed on 4 October 2023) and the 10 conserved motifs are color-coded (left panel). The a and a’ domains that are homologous to the TRX domain contain motifs 1 and 4, where the -CXXC- catalytic tetrad is included. The scale at the bottom indicates amino acid numbers of PbPDIs. The gene structure of the *PbPDI* is presented in the right panel. The yellow box indicates exons, black line introns, and the green box indicates the 5′ and 3′ untranslated regions (UTRs). The scale at the bottom indicates the nucleotide numbers of *PbPDIs*. CDS, coding sequence.

We investigated the exon–intron structure, 5′ and 3′ untranslated regions, and coding sequences of the 24 *PbPDIs* (Table 1, Figure 3). This shows that they all contain introns, where the maximum of 14 was found in Group VIII PDIs and the minimum number of 3 was found in groups VI and X PDIs. Eight *PbPDIs* have 3, four have 9, three have 4 or 11 each, and two have 8 or 10 or 14 introns each, respectively.

Further analysis of the structure of the PbPDI proteins was carried out by MEME to detect conserved motifs (Figure 3). Altogether, ten conserved motifs were identified, where Motif 1 and Motif 4 were present in groups I, II, and V PbPDIs and PbPDI4-2, Motif 2 in all groups except group IX, while Motif 5, Motif 6, and Motif 7, which may be related to the unique PAPS reductase domain, were only found in group XI PbPDIs.

### 3.4. Gene Duplication and Collinearity Analysis

**Figure 4:**
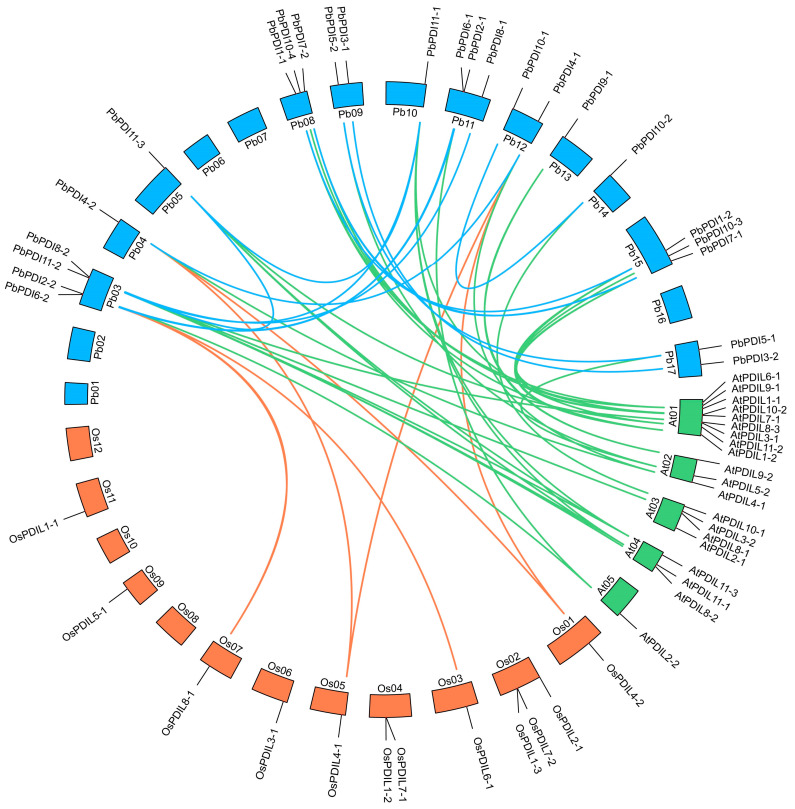
Collinearity analysis of the *PDI* genes of *P. betulaefolia*, *A. thaliana*, and *O. sativa* L. The chromosomes of the three species are represented by different colored boxes, with blue, green, and orange representing the chromosomes of *P. betulaefolia*, *A. thaliana*, and *O. sativa* L., respectively. The duplicated *PbPDI* genes on the chromosomes of *P. betulaefolia* are depicted by blue lines.

We performed a collinearity analysis of the 24 *PbPDIs* and found 12 *PDIs* have fragment repeats in the genome of ‘duli’ pear, i.e., homologous pairs were discovered, such as *PbPDI1-1* and *PbPDI1-2*, *PbPDI2-1* and *PbPDI2-2,* and others (Figure 4). However, no tandem duplications were observed in any of the 24 *PbPDIs*. Collinearity analysis was performed on the *PDI* gene family of *P. betulaefolia*, *A. thaliana*, and *O. sativa* L. This resulted in the identification of 27 pairs of homologous genes between *P. betulaefolia* and *A. thaliana* (green lines, Figure 4), whereas there were only 6 pairs between *P. betulaefolia* and *O. sativa* L. (orange lines, Figure 4).

The PbPDI proteins showed high sequence similarity within each group (Table S1). To assess the extent and type of selective pressure exerted on the members of the fragment repeats in the *PbPDIs*, we calculated the Ka/Ks ratio for each pair of paralogous genes. The Ka/Ks values of all duplicate gene pairs were <1, indicating that these genes underwent strong purification selection and changed slightly after repetition. The estimated time of differentiation of the *PbPDIs* shows that the duplication began 83.11 MYA and continued until 3.88 MYA (Table 3).

### 3.5. Analysis of Stress and Hormone Response Cis-Acting Elements in the Promoter Region of PbPDI Genes

**Figure 5:**
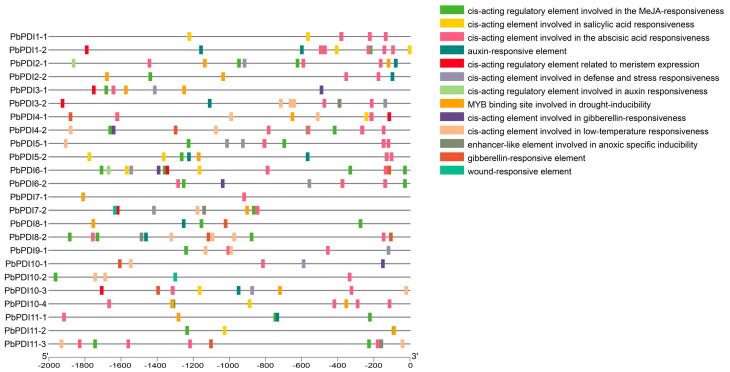
Prediction of cis-acting elements in the promoters of the *PbPDI* genes. The 2000 bp DNA sequences upstream of the coding regions of the 24 *PbPDIs* were analyzed. Different elements are color-coded (panel on the right) and their positions and sizes are indicated by the scale line below.

Using PlantCare, a number of possible plant hormone and stress response cis-acting elements in the promoters of the *PbPDI* gene family were identified. Among them, 21 are abiotic stress response cis-acting elements including ABA response elements, 16 are involved in drought stress, 11 in low-temperature stress, 10 in defense and stress, and 2 in wound response (Figure 5). Future expression study will verify these predictions.

### 3.6. PbPDIs Expression Profiles under Salt Stress

**Figure 6:**
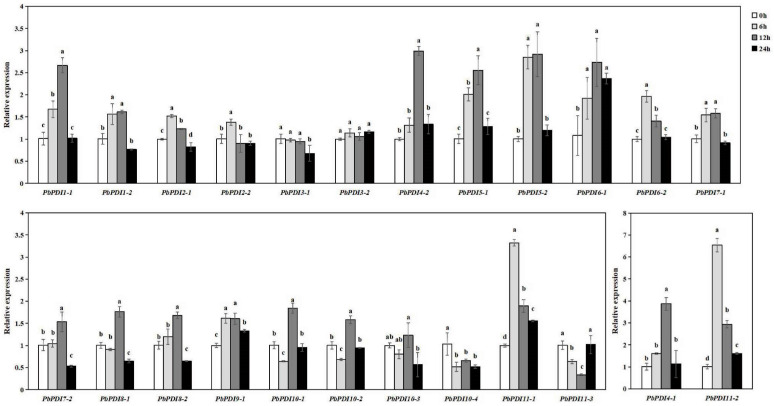
*PbPDIs* were upregulated under salt stress. Real-time PCR was carried out and the relative expression level of each gene was calculated using the 2^−∆∆Ct^ method, with *PbActin* as internal control and the transcript level at time 0 of individual *PbPDI* as 1. Each error bar represents standard error of the mean transcript of three replicates. Different letters represent significant differences at the 0.05 level after calculation using Duncan’s new multiple range method.

To further investigate the role of the *PbPDI* genes in salt stress, we analyzed the transcript levels of all the 24 *PbPDI* genes in ‘duli’ tissue cultured seedlings before and after treatment with NaCl at different time points of 0, 6, 12, and 24 h. As shown in Figure 6, most of the *PbPDI* genes were upregulated under salt stress. The expression levels of six of the 24 *PbPDI* genes (*PbPDI1-1*, *PbPDI4-1*, *PbPDI4-2*, *PbPDI5-1*, *PbPDI5-2*, *PbPDI6-1*, *PbPDI11-1*, *and PbPDI11-2*) were 2.6–6.5-fold higher at different time points of NaCl-treated than the control samples (0 h). Fifteen of the 24 *PbPDI* genes (*PbPDI1-2*, *PbPDI2-1*, *PbPDI2-2*, *PbPDI6-2*, *PbPDI7-1*, *PbPDI7-2*, *PbPDI8-1*, *PbPDI8-2*, *PbPDI9-1*, *PbPDI10-1*, *PbPDI10-2*, *and PbPDI10-3*) were expressed at levels 1.23–1.96-fold higher than control (0 h) at different time points (Figure 6).

Among the upregulated *PbPDI* genes, the transcription of four genes (*PbPDI6-1*, *PbPDI9-1*, *PbPDI11-1*, and *PbPDI11-2*) were significantly higher at all time points after salt treatment than those of the 0 h control, and seven genes (*PbPDI1-1*, *PbPDI1-2*, *PbPDI2-1*, *PbPDI5-1*, *PbPDI5-2*, *PbPDI6-2*, and *PbPDI7-1*) were significantly higher than the 0 h control at most time points. Although the expression of *PbPDI7-2*, *PbPDI8-1*, *PbPDI8-2*, *PbPDI10-1*, *PbPDI10-2*, and *PbPDI10-3* did not change after 6 h, they were significantly upregulated after 12 h of salt treatment. The expression of *PbPDI2-2* was significantly upregulated only after 6 h of salt treatment (1.4-fold increase) compared to those in the control group. In contrast, the expressions of three genes (*PbPDI3-1*, *PbPDI10-4*, and *PbPDI11-3*) were significantly downregulated under salt stress. The transcript of *PbPDI10-4* was significantly downregulated after 6 h of salt stress (1.9-fold decrease compared to the control). The expression level of *PbPDI3-1* decreased significantly after 24 h of salt stress (1.5-fold), while that of *PbPDI11-3* decreased at 6 and 12 h of salt stress but increased at 24 h (double that of the control). However, the expression of *PbPDI3-2* did not change significantly at all three time points compared to the control.

## 4. Discussion

We identified 24 members of the *PbPDI* gene family from ‘duli’ pear. Their predicted protein lengths differed considerably, indicating that their functions may be different. This is also supported by their different subcellular localization predicted (Table 1). The isoelectric point of 66% of the PDI proteins was <7, indicating that they are biased towards acidic amino acids. Phylogenetic analysis confirmed that the PbPDI proteins could be divided into four branches and eleven classes (Figure 1A). Both clades 1 and 2 contained PDI proteins with only one active TRX domain, and close phylogenetic relationships between members of the same clade may indicate that members in groups III and VII evolved from those in group I or II, resulting in the loss of an active domain. Similarly, group VI may have evolved from group IV or V members. The domain compositions between the PbPDI protein groups were significantly different (Figure 1C). This suggests that the *PbPDI* genes had undergone differentiation during evolution. Unlike other groups, members of groups VIII and X have nonspecific active sites, CYWS and CPFS (Table 2), instead of the typical -CXXC- motif [40,41]. These changes are likely to affect the redox potential, thereby affecting the function of these PbPDIs. In addition, the a’-type domain of PbPDI4-1 lacks the conserved arginine. The conserved arginine is believed to regulate the redox potential of the active site by regulating the pKa of the cysteine residue in the catalytic tetramer, whereas the conserved Glu–Lys pair is responsible for the proton transfer reaction, which is essential for the catalytic function of the TRX domain [42]. The a-type domain of PbPDI4-1 lacks the -CXXC-motif as well as the conserved lysine (Figure 2, Table 2). The lack of lysine leads to the inability to form the Glu–Lys pair. All of these changes are unique to PbPDIs and may affect the redox potential and, thus, their functions in ‘duli’ pear.

The PbPDI genes closely related in phylogeny tend to have similar exon–intron structures and motif distributions, but there were large differences between the groups. For example, members of group I (*PbPDI1-1* and *PbPDI1-2*) contain 9 introns, members of Group II (*PbPDI2-1* and *PbPDI2-2*) contain 11 introns, and members of group X (*PbPDI10-1* to *PbPDI10-4*) contain 3 introns. The motif distribution of the *PbPDI* genes follows the same rule. In addition, Motif1 and Motif4 contain the -CXXC- catalytic triad, which is critical for isomerase and redox activities. Among these, group IV (*PbPDI4-1* and *PbPDI4-2*) has two active TRX domains, but PbPDI4-1a does not have Motif1, possibly because the -CXXC- motif was lost during evolution, which is consistent with the results shown in Figure 1B. These rules indicate that PDI genes in the same group may have similar functions, and that PbPDI genes may have evolved functional diversity.

Gene duplication plays an important role in the expansion of gene family members during evolution [43,44]. We detected 12 duplicate gene pairs in the *PbPDI* family, including *PbPDI1-1* and *PbPDI1-2*, *PbPDI2-1* and *PbPDI2-2*, and *PbPDI3-1* and *PbPDI3-2*. These gene pairs are all fragment repeats with similar motif compositions and exon–intron structures, possibly originating from the same gene, leading to the continuous expansion of the PDI family in ‘duli’ pear. Collinearity analysis was performed on the *PDI* gene family of ‘duli’ pear, Arabidopsis, and rice (Figure 4), revealing that the evolutionary distance between ‘duli’ pear and the dicotyledon model plant Arabidopsis was shorter than that between ‘duli’ pear and the monocotyledon model plant rice, which is similar to previous studies [16].

When plants are under stress, such as salt, high temperature, and drought, proteins in the ER can undergo folding, misfolding, and aggregation, affecting normal cell function. PDIs play roles in correcting these unfavorable changes and ensure the reconstruction of active proteins from modified or misfolded proteins [45,46,47]. We found that 21 of the 24 *PbPDIs* in ‘duli’ pear seedlings were significantly upregulated when treated with NaCl (Figure 6). Consistent with this, we also identified cis-acting elements in the promoter regions of the majority of the *PbPDIs* that are involved in stress (Figure 5). Therefore, our combined results support the notion that PbPDIs play positive roles in salt tolerance in ‘duli’ pear.

In summary, we identified 24 *PbPDI* genes from the genome of ‘duli’ pear that share high homology with PDIs from Arabidopsis and other plant species. Their expression profiles in salt-treated samples were analyzed, showing that most of the *PbPDIs* were upregulated upon salt treatment. These preliminary data lay a foundation for further functional study of the PbPDI proteins and identification of potential candidate genes for breeding salt-tolerant ‘duli’ pear rootstock in the future.

## Figures and Tables

**Table 1:** The basic information of the 24 identified *PDI* genes and their encoded proteins of ‘duli’ pear. Chr, chromosome; MW, molecular weight; PI, isoelectric point; ER, endoplasmic reticulum; PM, plasma membrane.

Gene Name	Locus Name	Chromosome			No. of Exons	Proteins			Subcellular Localization
		**No.** **(** **Strand** **)**	**Length (bp)**	**ORF** **(bp)**		**Length (aa)**	**MW** **(kDa)**	**PI**	
*PbPDI1-1*	GWHPAAYT052317	Chr8+	3792	1503	10	500	56,1	4.92	ER
*PbPDI1-2*	GWHPAAYT023135	Chr15+	4157	1503	10	500	56.1	4.9	ER
*PbPDI2-1*	GWHPAAYT007464	Chr11-	4107	1716	12	571	63.5	4.71	Vacuole membrane
*PbPDI2-2*	GWHPAAYT035228	Chr3-	4261	1719	12	572	63.7	4.6	ER
*PbPDI3-1*	GWHPAAYT055473	Chr9-	4031	1275	11	424	47.2	5.17	Chloroplast
*PbPDI3-2*	GWHPAAYT029967	Chr17-	2865	1305	10	434	48.4	4.96	Chloroplast
*PbPDI4-1*	GWHPAAYT012267	Chr12+	21,142	1005	10	334	36.9	5.26	Extracellular
*PbPDI4-2*	GWHPAAYT039863	Chr4+	3528	1089	11	362	39.8	5.69	Extracellular
*PbPDI5-1*	GWHPAAYT028894	Chr17+	2992	1305	9	434	47.4	5.29	Vacuole membrane
*PbPDI5-2*	GWHPAAYT054427	Chr9+	2957	1308	9	435	47.7	5.38	ER
*PbPDI6-1*	GWHPAAYT007378	Chr11+	3446	450	4	149	16.7	4.66	Extracellular
*PbPDI6-2*	GWHPAAYT035173	Chr3+	2815	450	4	149	16.8	4.89	Extracellular
*PbPDI7-1*	GWHPAAYT024090	Chr15+	4405	1329	5	442	49.8	5.01	PM
*PbPDI7-2*	GWHPAAYT053038	Chr8+	3456	1332	5	443	49.8	4.9	PM
*PbPDI8-1*	GWHPAAYT008875	Chr11+	9376	1443	15	480	53.5	7.36	PM
*PbPDI8-2*	GWHPAAYT036573	Chr3+	8640	1443	15	480	53.7	7.06	PM
*PbPDI9-1*	GWHPAAYT013244	Chr13-	4914	1536	12	511	57.1	8.24	Chloroplast
*PbPDI10-1*	GWHPAAYT009923	Chr12+	4996	891	4	296	33.7	9.8	PM
*PbPDI10-2*	GWHPAAYT016488	Chr14+	4312	900	4	299	33.9	9.73	PM
*PbPDI10-3*	GWHPAAYT023589	Chr15-	2655	942	4	313	34.9	9.07	Cytoplasm
*PbPDI10-4*	GWHPAAYT052662	Chr8-	2739	810	4	269	30.0	8.21	PM
*PbPDI11-1*	GWHPAAYT005858	Chr10+	3378	1398	4	465	51.6	6.36	Chloroplast
*PbPDI11-2*	GWHPAAYT044219	Chr3-	2551	1395	5	464	51.6	6.74	Chloroplast
*PbPDI11-3*	GWHPAAYT036474	Chr5+	2744	1383	4	460	50.8	8.9	Chloroplast

**Table 2:** Structural and functional characteristics of the 24 PbPDI proteins of ‘duli’ pear (*P. betulaefolia*).

Name	Signal Peptide	Trans- Membrane	Domain Organization	Active Site Motif	Conserved Charge Pair Sequence	Conserved Arginine	C-Terminal Signal
PbPDI1-1	1-24	No	a-b-a’	CGHC, CGHC	E54-K88, E398-K431′	R128, R468	KDEL
PbPDI1-2	1-24	No	a-b-a’	CGHC, CGHC	E54-K88, E398-K431	R128, R468	KDEL
PbPDI2-1	1-23	No	a-b-a’	CGHC, CGHC	E113-K145, E452-K485	R181, R523	RFEG
PbPDI2-2	1-23	No	a-b-a’	CGHC, CGHC	E113-K145, E452-K485	R181, R523	KDEL
PbPDI3-1	1-31	No	a-c-b	CSRS	L105-K139	F175	SSAQ
PbPDI3-2	1-29	No	a-b	CARS	L103-K137	F173	ACIL
PbPDI4-1	1-23	No	a^o^-a-D	CGHC	E138-N171	R90	TSSS
PbPDI4-2	1-23	No	a^o^-a-D	CGHC, CGHC	E47-K80, E166-N199	R118, R237	ASSS
PbPDI5-1	1-24	No	a^o^-a-b	CGHC, CGHC	E54-A85, E182-H213	R122, R251	KEEL
PbPDI5-2	1-25	No	a^o^-a-b	CGHC, CGHC	E55-A86, E183-H214	R123, R252	KDEL
PbPDI6-1	1-27	No	a	CKHC	K51-E84	R121	DKEL
PbPDI6-2	1-27	No	a	CKHC	K51-E84	R121	DKEL
PbPDI7-1	1-30	375-397	a-c-b-t	CGHC	D61-K95	R131	EKED
PbPDI7-2	1-30	376-398	a-c-b-t	CGHC	D62-K96	R132	EKED
PbPDI8-1	No	442-464	g-a-f-t	CYWS	N163-K202	R248	GKNF
PbPDI8-2	No	442-464	g-a-f-t	CYWS	N163-K202	R248	GKNF
PbPDI9-1	1-18	475-511	a-g-t	CPAC	K71-R109	Q149	RSWS
PbPDI10-1	1-22	No	a	CPLS	L93-I120	K152	SSTN
PbPDI10-2	1-22	195-217	a-t	CPFS	L94-I121	K153	SLSS
PbPDI10-3	1-27	No	a	CPFS	L85-F116	R152	YSSD
PbPDI10-4	1-22	215-237	a-t	CPFS	L85-F116	R152	SAVV
PbPDI11-1	No	No	h-a′	CQFC	V379-K411	R452	NALR
PbPDI11-2	No	No	h-a′	CPFC	V377-K410	R451	NALR
PbPDI11-3	No	No	h-a′	CRFC	V374-K406	R447	NALR

**Table 3:** Estimated Ka/Ks ratios of the segmentally duplicated *PbPDI* genes with their divergence time in ‘duli’ pear (*P. betulaefolia*).

Duplicated Gene Pairs		Ka	Ks	Ka_Ks	Duplication Type	Types of Selection	Time (MYA)
*PbPDI1-1*	*PbPDI1-2*	0.030656727	0.228689663	0.134053839	Segmental	Purifying selection	7.62
*PbPDI2-1*	*PbPDI2-2*	0.037174043	0.264643094	0.140468594	Segmental	Purifying selection	8.82
*PbPDI3-1*	*PbPDI3-2*	0.080273354	0.281013353	0.285656725	Segmental	Purifying selection	9.37
*PbPDI4-1*	*PbPDI4-2*	0.053035942	0.128765197	0.411881031	Segmental	Purifying selection	4.29
*PbPDI5-1*	*PbPDI5-2*	0.017175359	0.240972829	0.071275086	Segmental	Purifying selection	8.03
*PbPDI6-1*	*PbPDI6-2*	0.041379614	0.306937951	0.134814266	Segmental	Purifying selection	10.23
*PbPDI7-1*	*PbPDI7-2*	0.05256445	0.158091931	0.332492932	Segmental	Purifying selection	5.27
*PbPDI8-1*	*PbPDI8-2*	0.02186113	0.223403215	0.097855039	Segmental	Purifying selection	7.45
*PbPDI10-1*	*PbPDI10-2*	0.044270767	0.116345503	0.380511196	Segmental	Purifying selection	3.88
*PbPDI11-1*	*PbPDI11-2*	0.022953656	0.301454658	0.076142981	Segmental	Purifying selection	10.05
*PbPDI11-1*	*PbPDI11-3*	0.163012376	2.493442125	0.065376443	Segmental	Purifying selection	83.11
*PbPDI11-2*	*PbPDI11-3*	0.154988455	2.21329614	0.070026081	Segmental	Purifying selection	73.78

## Data Availability

The original contributions presented in the study are included in the Article/Supplementary Materials, further inquiries can be directed to the corresponding authors.

## Supplementary Materials

The following supporting information can be downloaded at: https://www.mdpi.com/article/10.3390/genes15080968/s1. Table S1. Sequence identification of 24 PDI proteins from ‘duli’ (unit %); Table S2. Mean value of FPKM three replicates of PDI family in each organ of ‘duli’; Table S3. Quantitative specific primers of PDI gene family in ‘duli’; Table S3. List of the PDI protein sequences used for phylogenetic analysis.
