# Supplementary material for: A Global Identification of Protein Disulfide Isomerases from ‘duli’ Pear (Pyrus betulaefolia) and Their Expression Profiles under Salt Stress
Source: Genes (Basel). 2024 Jul 23;15(8):968. doi: 10.3390/genes15080968 (PMC11353384; doi:10.3390/genes15080968)
Supplement: Supplementary file 1 [file genes-15-00968-s001.zip › genes-3061069-supplementary.pdf]

Supplementary Materials:

Table S1. Sequence identification of 24 PDI proteins from Pyrus betulaefolia (unit %)

protein	A	B	C	D	E	F	G	H	I	J	K	L	M	N	O	P	Q	R	S	T	U	V	W	X
PbPDI1-1(A)	100	**96**	43	47	35	32	20	27	32	36	16	16	34	40	22	23	29	21	21	19	15	26	30	32
PbPDI1-2(B)		100	42	43	35	35	22	26	32	35	16	16	34	39	20	25	29	21	22	20	14	23	23	27
PbPDI2-1(C)			100	**96**	37	37	27	26	23	23	11	13	30	35	26	21	31	9	21	16	14	31	32	23
PbPDI2-2(D)				100	36	37	26	24	23	21	11	13	29	31	25	24	30	10	10	20	21	31	32	24
PbPDI3-1(E)					100	**81**	22	29	32	29	15	12	36	32	27	30	30	24	29	31	24	11	10	10
PbPDI3-2(F)						100	25	29	30	30	15	15	29	30	27	26	23	24	28	27	24	10	11	9
PbPDI4-1(G)							100	**86**	39	38	19	20	21	17	29	29	23	23	23	22	27	19	19	17
PbPDI4-2(H)								100	44	44	20	20	31	26	25	24	32	22	18	29	26	14	17	8
PbPDI5-1(I)									100	**99**	16	16	31	30	29	27	16	21	19	28	24	16	16	16
PbPDI5-2(J)										100	16	16	30	31	29	27	22	20	20	27	24	16	16	16
PbPDI6-1(K)											100	**97**	16	15	13	13	12	21	19	15	21	12	12	12
PbPDI6-2(L)												100	15	15	15	15	13	20	20	18	9	11	11	11
PbPDI7-1(M)													100	**94**	22	22	31	27	26	25	19	7	7	8
PbPDI7-2(N)														100	26	21	31	28	28	25	18	8	8	8
PbPDI8-1(O)															100	**98**	21	23	24	30	19	13	13	14
PbPDI8-2(P)																100	22	24	25	30	22	13	13	16
PbPDI9-1(Q)																	100	21	21	25	18	7	9	8
PbPDI10-1(R)																		100	**93**	51	39	9	10	19
PbPDI10-2(S)																			100	50	38	9	9	7
PbPDI10-3(T)																				100	78	10	12	12
PbPDI10-4(U)																					100	10	13	13
PbPDI11-1(V)																						100	**97**	**83**
PbPDI11-2(W)																							100	**83**
PbPDI11-3(X)																								100

Table S2. Quantitative specific primers of PDI gene family in *Pyrus betulaefolia*

Primer Name	Sequence(5' to 3')
PbPDI1-1-F	CAAGACTACTGACGATGCAACTAC
PbPDI1-1-R	GATGAATCACCACGAGGAAGG
PbPDI1-2-F	CGTCCTCACTCTCGACAGCTC
PbPDI1-2-R	CCTCTTCATTGGCATCAACC
PbPDI2-1-F	CACGGAGGAGAACGAGCTGTC
PbPDI2-1-R	CACCAGAGAGTTGAGGTAGCCC
PbPDI2-2-F	GAGCTGTCGCAGGAGTACGGC
PbPDI2-2-R	CAGCAGTCAATATGCGTTCCGC
PbPDI3-1F	CTGTTTGTCAATGGCACCTCC
PbPDI3-1R	CGAACTGGATGGCACTGTCAG
PbPDI3-2F	AGACAGGTTCTTAGAGCGCC
PbPDI3-2R	AGCGCCTTATCGAGTTTCCC
PbPDI4-1-F	GAAGGAGGTCGGTCAAGATC
PbPDI4-1-R	CATTGAATTGTGGGATAGCCGG
PbPDI4-2-F	GTCGAGTTCTACGCTCCCTG
PbPDI4-2-R	GCAGTACGGGCACCTTCATAC
PbPDI5-1-F	GAGAGAGAGAGAATGAGAGAGAATC
PbPDI5-1-R	GGTGTAAGAGCCTGACAATGTCC
PbPDI5-2-F	CTGATTCGCACAACAAATCTCAG
PbPDI5-2-R	GTTAGAGCCTGACAATGCCCG
PbPDI6-1-F	CGCTGACACCTTCTCCGAC
PbPDI6-1-R	CTCGTACTACAATCAACCTCCC
PbPDI6-2-F	CCTGACAACCCATACCCAAG
PbPDI6-2-R	GCGACTTCTTCACCATCATAG
PbPDI7-1-F	GATGAGGATGAAGAGAAGGC
PbPDI7-1-R	GAACTGTGAGACTTGAGATGCC
PbPDI7-2-F	CTTGGACCTGGTCTTGGTTG
PbPDI7-2-R	CCATTGTATTCCATCGGAACAC
PbPDI8-1-F	CTCCGGCAAGATCAAGTCTG
PbPDI8-1-R	CAGATGTAGAGGTGCTGACGG
PbPDI8-2-F	CGTGAGTGATGTGTTGGGAAC
PbPDI8-2-R	CCAGAATACTCATCTCCATCCC
PbPDI9-1-F	CTGACAGGCAGGGAGACCAA
PbPDI9-1-R	CAGAAGAACCCATAACCCACAG
PbPDI10-1-F	CCGTTACAGCCTTCAATCTC
PbPDI10-1-R	GAGGGAACATGAAGCTGAGC

PbPDI10-2-F	GGTACAGCCTTCAATCTCAATG
PbPDI10-2-R	GAGGGAACATGAAGCTGAGC
PbPDI10-3-F	GTCAACGGCGCTTCCAGATC
PbPDI10-3-R	CTTGGCCTGATGGCTGATTC
PbPDI10-4-F	GCTTGCAGATCCGTTGAATC
PbPDI10-4-R	GATGGCTGATTCTTCAAATGC
PbPDI11-1-F	TTGAGGTGGAGGACTATGCTC
PbPDI11-1-R	CAGCCTCCCAGTATCCAAGC
PbPDI11-2-F	AGAGGTGGAGGACTATGCGC
PbPDI11-2-R	GCCGTAGTGCTTCTCTACTGTG
PbPDI11-3-F	GGGAGTGCTAATGTCCCAG
PbPDI11-3-R	CCCATCAGCTCTGAACTTACC

Table S3. List of the PDI protein sequences used for phylogenetic analysis

Name	Accession	Sequence
PbPDI1-1	GWHPAAYT052317	MASSSRVSLCFFFLLAVFSTAIRAEEAESKEYVLPLDSSNFADTVGKHDFIVVEFYAPWCGHCKKLAPEYEKAASILSKNDPPVILAKVDANEEANKGLASDYEVKGFPTIKILRNGGKTIQEYKGPREAEGIVEYLKKQSGPPSVEIKTTDDAITLVGDKKIVVVGVFKEFSGEAYNNFSALAEKLRSDYEFGHTLDAKLLPHGDSSVSGPVVRLFKPFDELFVDIEDFHVDALEKFVEDASVPLVTEFNNDQTNHPFVIKFFNSPNEKAMLFLNFSDESSDAFKSTYREVAEKYKKEGISFLIGDLEASQGAFQYFGLKEDQVPLIIIQTPGGQKFLKPNLQPDHITSWVKEYKDGKVSPFKKSEPIPEQNNEPVKVVVADSIQDYIKSEKNVLLEFYAPWCGHCKKLAPILDEVAASYEKDSDVVIAKFDATANDVPSDFDVKYYPTLYFKTASGKVLSYDEEDRTKEAIIAFIEKNRDKIEKQAESEKQESGKDEL
PbPDI1-2	GWHPAAYT023135	MASSSRVSLCFFLLLAVFATAIRAEEAESKEYVLTLDSSNFDDTVSKHDFIVVEFYAPWCGHCKKLAPEFEKAASILSKNDPPVILAKVDANEEANKGLASDYEVKGFPTIKILRNGGKTIQEYKGPREAEGIVEYLKKQSGPASAEIKTTDEATTLVGDKKIVIVGVFKEFSGEVYNNFSALAEKLRSDYEFGHTLDAKLLPRGDSSVSGPVVRLFKPFDELFVDFEDFHVDALEKFVEEASLPVITEFNNDPTNHPFVVKFFNNPNEKAMLFLNFSGESSDAFKSTYREVAEKYKKEGISFLIGDLEASQGAFQYFGLKEDQVPLIIIQTPDGQKFLKPNLQPDHITSWVMEYKDGKVSPYKKSEPIPEPNNEPVKVVVADNIQEYIKSGKNVLLEFYAPWCGHCKKLAPILDEVAVSYEKDSDVVIAKFDATANDVPSDFDVKYYPTLYFKTASGKVLSYDEEDRTKEAIAAFIEKNRDKIEKQVEAEKQESGKDEL

PbPDI2-1	GWHPAAYT007464	MASRLVLAFALSALLFFSHSVLCKDSHPTDDDDEDLSFLEEPTDHGDAAPHYPDSDHYDE<br>DNLDDLENYSDFDEGGDHEDSYKQPEVDEKDVVVLKAANFSDAVEKNRFMMVEFYAP<br>WCGHCQALNPEYAAAATELKGEDVVLAKVDATEENELSQEYGVEGFPTIFFFIDGVHKPY<br>TGQRTKEGIVTWIKKKIGPGIQNVTTLEGAERILTAESKVVLGYLNSLVGPESDELAAASR<br>LEDEVTFYQTVDPKVAKLFHLDAEVKRPALVLLKKEAEKLSYFDGKFDKSAIAEFVFANK<br>LPLVITFTRDNAPQIFESTIKKQLLLFATSKDSEKSLPEFQKAAQLFKGKLIFVYVETDNEDI<br>GKPVSDYFGVTSEAPTVLGYTGNDDGRKFVLDGEVTLANLKTFGEDFIEDKLKPFYKSDPI<br>PETNDGDVKIVVGNNFDDIVLDESKDVLLEIYAPWCGHCQSLEPTYNKLAKHLRGIDSIVI<br>AKMDGTTNEHPRGKADGFPTLLFFPAGNKSFDPIPVDCDRTVVAFYKFLKKNASIPFKLQ<br>KPASTPKSEGSAATESQGSSSRRRRFEG
PbPDI2-2	GWHPAAYT035228	MASRLVLAFALSALLFFSHSVFCKESHPIDDDDEDLSFLEEPTDHGDDAPHYPDPDHYDED<br>NYDDLENYSDFDEGGDHEDSYKEPEVDEKDVAVLKAANFSDVVEKNRFVMVEFYAPWC<br>GHCQALKPEYAAAATELKGEDVILAKVDATEENELSQEYGIEGFPTIFFFIDGVHKPYAGQ<br>RTKEGIVTWIKKKIGPGIQNVTTLEDAERILTAESKVVLGYLNSLVGPESDELAAASRLEDE<br>VSFYQTVDPKVAKLFHLDAEVKRPALVLLKKEAEKLSYFDGKFDKTAIVEFVFANKLPLV<br>ITFTRDNAPQIFESTIKKQLLLFASSKDSEKVLPDFQKAAQLFKGKLIFVYVETDNEDIGKP<br>VSDYFGVTSEAPTVLGYTGNDDGRKFFLDGEVTLANLKAFGEDFIEDKLKPFYKSDPIPES<br>NDGDVKIVVGNNFDEIVLDESKDVLLEIYAPWCGHCQSLEPTYNKLAKHLRGIDSIVIAK<br>MDGTTNEHPRAKADGFPTLLFFPAGNKSFDPITVDSDRTVVAFYKFLKKNASIPFKIQKPA<br>STPKSEGSAATESKGSGSAEDSKDEL
PbPDI3-1	GWHPAAYT055473	MSRSKPTSRFIIFVLTLILTLPLHFPTPIKSSEPQNLTADETEDGELEELLALDQQVKQEEDQ<br>DGAPTTRSLEAELLSNAQRIVLELNHDNTKRVIENNEFVLVLMYAPWCSRSAELMPLFAE<br>AATALKELGSPLLMAKIDAERHTKAASSLEIKGFPTLLLFVNGTSQAYTGGFSAKEIVIWA<br>RKKTGEPVIRINSSDIFVVVLFEKFEGPEHKEFVKAATADNAIQFVEASNIEVANILFPNMK<br>PTKGTFRADEILQFLDHNKFPLVNRLTEANSAKVYSSPIQIQVHVFADADDFKKLLEPLQD<br>VARQFKSKILFIYIDITDGNLAKPYLTVFGLEESNSTVRSSKYLLESDPTQSNLEEFCSGLLQ<br>GTVSPHFKSQLIPDNKNETVLSVVGKTLDDLVLNSHKNVVLEPSPHSKSSSSAQ
PbPDI3-2	GWHPAAYT029967	MSRPKPTSRFILFTLTLLLLLHFPTPIKSSEPQNPAADETEDGELEELLALDEQVEQEEDQD<br>GGPATRSSEAELLSKAQRIVLELNHDNTKRVIENNEYVLVLGYAPWCARSAELMPQFAEA<br>ATALKELGSPLLMAKLDAERHAKTASSLEIKGFPTLLLFVNGTSQAYTGGFSAQAEFLKKS<br>DIFVFGLFENFEGPEHKEFVKAAAADSAIQFVEVSNIEVANVLFPNVKPTNVFLGIVKSEPE<br>RYTAYEGTFRVDEILQFLDYNKFPLVNRLTEANSAKVYSSPIQLQVHVFADADDFKKLLE<br>PLQDVARQFKSKILFIYIDITDENLAKPYLTVYGLEESNSAVVTAFDIRGNSKYLLESNPTPS<br>NLEEFCSGLLHGTVSPHFKSQPIPHNKNVTVQSIVGKTFDDLVLNSHKNVVLENKKVNEL<br>YHACIL
PbPDI4-1	GWHPAAYT012267	MVSSKTLFAFGALALLLASSVYADDVVVLTEDNFEKEVGQDRAALVEFYAPWLIVMSTR<br>AVCGKYGVSGYPTIQWFPKGSLEPKKYEGGRTAEALAEFVNKEGGTNVKLAVAPSHVVV<br>LTQDNFNEVVLDETKDVLVEFYAPWCGHCKSLAPTYEKVASAFNLEDGVVIANLDADKY<br>KDLAEKYGVSGFPTLKFFPKNNKEGEEYGDGRDLDDFVKFINEKSGTSRDGKGQLTSKAG<br>ILENLDELVKEFVKSSNDEKKAIFSKIEEEVGKLEGSAARYGKIYLKAAKNSLEKGPDYAK<br>NEIQRLGRILEKSVNPTKADEFTLKKNILYTFTSSS

PbPDI4-2	GWHPAAYT039863	MASSKTLIAFGALALLFASSAFADDVVVLTEDNFEKEVGQDRAALVEFYAPWCGHCKKL<br>APEYEKLGSSFKKAKSILIAKVDCDEHKGVCGKFGVSGYPTIQWFPKGSLEPKKYEGART<br>AEALAEFVNKEGGTNVKIAADPSHVVVLTQDNFNEVVLDETKDVLVEFYAPWCGHCKSL<br>APTYEKVASAFKLEDSVVIANLDADKYKDLAEKYGVSGYPTLKFFPKNKKEGEEYGGGR<br>DLEDFVAFINEKSGTSRDAKGQLTSKAGIVENLDELVKEFVKSSNDEKKAIFSKIEEEVGK<br>LEGSAARYGTIYLKAAKKSLEKGADYAKNEIQRLERILEKSVNPTKADEFTLKKNILYTFA<br>SSS
PbPDI5-1	GWHPAAYT028894	MRENQFRAVSIIFLFFFVVSVCDALYGPSSPVLQLTPSNFKSKVLDSNRVVLVEFFAPWCG<br>HCQALTPIWEKAATVLKGVAAVAALDADAHKSLAQEYGIRGFPTIKVFVPGKPPVDYQG<br>ARDVKPIAEFALQQIKALLKDRLSGKTTGGSSEKSEPSASVELNSQNFNELVLKSKDLWIV<br>EFFAPWCGHCKKLAPEWKKAAKNLQGKVKLGHVDCDAEKSLMSKFNVQGFPTILVFGA<br>DKDSPLPYEGARSASAIESFALEQLETNVAPPEVTELSGPDVMEEKCGSAAICFVAFLPDIL<br>DSKAEGRNKYIQQLLSVAEKFKRSPYSFVWAAAGKQPDLENRVGVGGYGYPALVALNA<br>KKGAYAPLKSAFEVDQITEFVKEAGRGGKGNLPLEGTPNIVKIEPWDGKDGEIIEEDEFSL<br>DELMGEDTTNKEEL
PbPDI5-2	GWHPAAYT054427	MRENQFRAVPIIFFLFFFVFNVCDALYGPSSPVLQLTPSNFKSKVLESNRVVLVEFFAPWCG<br>HCQALTPIWEKAATVLKGVATVAALDADAHKSLAQEYGIRGFPTIKVFVPGKPPVDYQG<br>ARDVKPVAEFALQQIKALLKDRLSGKTTGGSSEKSEPSASVELNSQNFNELVLKSKELWIV<br>EFFAPWCGHCKKLAPEWKKAAKNLQGKVKLGHVDCDAEKSLMSKFNVQGFPTILVFGA<br>DKESPLPYEGARSASAIESFALEQLETNVAPPEVTELTGPDAMEEKCGSAAICFVAFLPDIL<br>DSKAEGRNKYIQQLLSVAEKFKRSPYSYVWAAAGKQPDLENRVGVGGYGYPALVALNA<br>KKGAYAPLKSAFEVDQITEFVREAGRGGKGNLPLEGTPSIVKIEPWDGKDGQILEEDEFSL<br>EELMGEDTTNKDEL
PbPDI6-1	GWHPAAYT007378	MKAGSASLVFISVLLFLLPILTTYTEAEVITLTADTFSDKVKEKDTAWFVKFCVPWCKHCK<br>NLGTLWEDLGKTMEGEEEIEVGEVDCSTSKPVCSKVDIHSYPTFKVFYDGEEVAKYQGPR<br>DVDSLKNFVLDEAEKAATKAQLDSDKEL
PbPDI6-2	GWHPAAYT035173	MKTHLASLVLISVLLFLLPILTTHTQAEVITLTADTFSDKVKERDTAWFVKFCVPWCKHCK<br>NMGTLWEDLGKTVEGEDEIEVGEVDCSTSKPVCSKVDIHSYPTFKVFYDGEEVAKYQGP<br>RDVDSLKNFVLEEAEKAATKAQLDNDKEL
PbPDI7-1	GWHPAAYT024090	MSTMRMRVRALCCWLLLILLTALDWPAVAASSSWTVDGTVLELDDSNFDSAISALDLVL<br>VDFYAPWCGHCKRLSPQLDAAAPLLAGLKHPVAIAKLNADKFTSVARKYEIDAYPTLKLF<br>MHGVPLEYNGPRKAESLVRYLKKFAAPDVSILESDSAISEFVEAAGTYFPIYIGFGLDESLIS<br>KLAIKYKKKAWFSVAKDFSEDVMVLYDFDKVPALASLHPTYDERNIFYGPFEEDFLEDFI<br>QQSLFPLAMPINYETLKSLSDDERKIVLTIVKDEDEEKAKKLIKILKSAASANRDFVFGYVG<br>IKQWEDFADTFGATKKTKLPKMVVWDRMEEYFTVNGSESIDEEDQASQVSQFLQGYKEG<br>RIIKKRIGGPSFGSVMSSFFGMRTVYIMVFLVIVTMLIRSISKEDDEPPVVRTADQIDPATSS<br>STEAESREHRSGEKED
PbPDI7-2	GWHPAAYT053038	MGRMIGMRVRPLWCWALLILLAALDSPAVAAVSSWAVDGTVLELDDSNFDSAISALDLV<br>LVDFYAPWCGHCKRLSPQLDAAAPLLAGLKHPVAIAKLNADKFTSLARKYEIDAFPTLKL<br>FMHGVPMEYNGPRKAESLVRYLKKFAAPDVSILESDSAISEFVQAAGTYFPIYIGFELNESL<br>VSKLAIKYKKKAWFAVAKDFSEDVMVLYDFDKVPALASLHPTYDERNIFYGPFEEEFLED<br>FIRQSLFPLAMPINYETLKSLSDDERKIVLTIVEDEDEEKSKKLINILKSAASANRDFVFGYV<br>GIKQWEDFADTFGVNKKTKLPKMVVWDRMEEYITVNGSESIDEEDQASQVSQFIQGYKE<br>GRIIKERIGGPSFASFMTSFIGIGTVYIIVFVVIVMMLIRSINKEDDESPVARTGDQVDRATSS<br>TVEAESKEHRSGEKED

PbPDI8-1	GWHPAAYT008875	MISSGKIKSVDFYRKIPRDLTEASLSGAGLSIVAALAMMFLFGMELNNYLTVSTSTSVIVDKSSDGDFLRIEFNISFPALSCEFASLDVSDVLGTNRLNITKTIRKFSIGTDLRPTGTEFHSGPVLHNIKHGDGDEFSGDGSISLTAQNFEKITHQHPILVVNFYAPWCYWSNRLKPSWEKAAKIIRERYDPEIDGRILMAKVDCTEEGDLCKRNHIQGYPSIRIFRKGSDVRDDHGHHEHESYYGDRDTDSLVKTMETLVAPIPVESEKLALEGKSNNGGENAKRPAPLTGGCRIEGYVRVKKVPGNLVISAHSGAHSFDASQMNMSHVISHFSFGRMIAPKVMSDVKRLVPYLGVSHDRLNGRSFINHRDLGANVTIEHYLQIVKSEVITGRSHKLIEEYEYTAHSSLAQSLQIPVAKFHFELSPMQVLITENPKSFSHFITNVCAIIGGVFTVAGIMDSILHNTIRMMRKVELGKNF
PbPDI8-2	GWHPAAYT036573	MISTGKIKSVDFYRKIPRDLTEASLSGAGLSIIAALAMMFLFGMELNNYLTVSTSTSVIVDKSSDGDFLRIEFNVSFPALSCEFASVDVSDVLGTNRLNITKTIRKFSIGTDLRPTGSEFRSGPVLHNIKHGDGDEYSGDGSISLTAQNFEKFTHQHPILIVNFYAPWCYWSNRLKPSWEKAAKIIRERYDPEIDGRILMAKVDCTEEVDLCRRNHIQGYPSIRIFRKGSDVRDENGHHEHESYYGDRDTDSLVKTMEALVAPIPVESEKLSLEDKSDNRGENAKRPAPLTGGCRIEGYVRVKKVPGNLVISAHSGAHSFDASQMNMSHVISHFSFGRMIAPKVMSDVKRLVPYLGGSHDRLNGRSFINHQDLGANVTIEHYLQIVKSEVITGRSHKLIEEYEYTAHSSLVQSLQVPVAKFHFELSPMQVLITENQKSFSHFITNVCAIIGGVFTVAGIMDSILHNTIRMMRKVELGKNF
PbPDI9-1	GWHPAAYT013244	MSPALRLLILSLFSLTSALSPASASSAAGSRSILRAVNDDNADVRDFAVDLNASSFDAVLSDTPATFAVVEFFAHWCPACRNYKPHYEKVARLFNGPDAVHPGMVLMTRVDCASKVNTKLCDKFSVGHYPMLFWGPPSKFVSAGWEPNQAKSVIRVIDDGRTADRLLSWINKQLGSSFSLDDQKFENEHISSNASDPEQIARAVYDVEEATSTAFEIILEHKMIKSKTRASLVKFLQLLVAHHPSRRCRKGSAEVLVNFDDLYPLDILAADRLGDQDVQAALQKFQICGKDVPRGYWMFCRGSKNDTRGFSCGLWVLLHSLSVRIEDGESNFVFTTICDFVHNFFVCEECRQHFYDMCSSVSSPFNKSRDLVLWLWSAHNKVNERLIKEESSLGTADPKFPKMIWPPRQLCPSCYLSSSQKNKEIDWHKDEVFKILTSYYGKTLVSLYKDKGIVGNDKISGALEDLVASTNALVVPLGAALAIAVASCAFGALACYWRSQQKSRKPRRSWS
PbPDI10-1	GWHPAAYT009923	MDKSVILLYIAALAASFRLVSSSALCPHESDFFRYSLQSQCPISISPHPPLKVDGNFLDRALASQQRTDYTAVLFYASWCPLSRTMYPTFEKLSFMFPQVEHLAIEQSSALPSVFSRYGIHSFPSILLVNQTSMVRYHGPKNLSSLAQFYQKTTGLEPVQYFDGDHTVSLNIREKSIIRSMSNMSLREISRREPFLAFATLFLCLRVLLYIFPKVLTRLQAFWVLYVPHFNLGIFGETSQIMGRILHMVDVRRIWTKLRLCKTRNFHEGAKNARVWASSLTSVSLGKSSSARSSTN
PbPDI10-2	GWHPAAYT016488	MDKSVIFLCIAALATSLRLVSSSSVLCPHDSDFFRYSLQSQCPIWISPHPPLKVDGNFLDRALASQQRTDYTAVLFYASWCPFSRTMYPTFEKLSFMFPQVEHLAIEQSSALPSVFSRYGIHSFPSILIVNQTSRVRYHGPKSLSSLAQFYQKTTGLKPVQYFDGDQIVSLNIREKSLIQSMGNMSLREISRREPYLAFAILFLCLRVLLYIFPKVLTRLHAFWVLYVPHFNLGIFGEASQSMGCILPMVDVRRIWTKLRLCKTRNFHEGAKNARVWASSLTSVSLGKSSSARSSSLSS
PbPDI10-3	GWHPAAYT023589	MGIRVWESGIVILVLWVRLISAAEPTSCPAESAADAIFGFRYSNCPVNGASRSVESMGVIVGDEVSLQRALNMVHKNTHDYVAVLFYASWCPFSRMFKPTFSILASLYPSIPHFAFEESAIRPSILSKYGVHGFPTLFILNSTTRIRYQGARTPGSLIAFYSHVTGIKTVSLDQLSLEKIGYPLNHEKHDSTEQESCPFSWARSPENLLRQETYLALASAFVLLRLLYSFYPALLSFVQSAWRQHIQNMRLGSLLEHPLAFLKRAVQLFNSLKEPCKRSNLQEGAMNARVWASKSLATVSIGDASTSRGYSSD

PbPDI10-4	GWHPAAYT052662	MGIRVWESGIVILVLWVRLVCAAEPASCPTESVANTIFGFRYSNCPVDGACRSVESMGVIVGDEVSLQRALNMVHKNTHDYVAVLFYASWCPFSRMFKPTFSILASLYPSIPHFAFEESAIRPSVLSKYGVHGFPTLFILNSTMRIRYQGARTPGSLIAFYSHVTGFKTVSLDQLSLEKIGYPLNHEKHDSTEQESCPFSWARSPENLLRQETYLALASAFVLLRLLYFFLPALLSFAQSAWRRHIQGHDIGELVGAPFGFSKAGSAVV
PbPDI11-1	GWHPAAYT005858	MALAVTSSSSASISGRTFTPSSEPKAPQLGSIRVFDRSYGQIQCHAVTFSERRSSVKPVNAQPKRSESIVPSAATVVAPEVVEEVEVEDYAQLARELENASPLEIMDKALEKFGNDIAIAFSGAEDVALIEYAHLGRPYRVFSLDTGRLNPETYQLFDTVEKHYGIRIEYMFPDAVEVQALVRTKGLFSFYEDGHQECCRVRKVRPLRRALKGLRAWITGQRKDQSPGTRSEIPVVQVDPVFEGLDGGAGSLVKWNPVANVEGDIWNFLRAMNVPVNSLHSKGYISIGCEPCTRSVLPGQHEREGRWWWEDAKAKECGLHKGNIKQEGGGENGKAAQSNGAATETDIFTSQNLVTLSRTGIENLAKLEDRKEPWIVVLYAPWCQFCQAMEASYVELADKLAGSGVKVGKFRADGEQKEFAQKELQLGSFPTILFFPKHSSQPIKYPTEKRDVNSLLAFVNALR
PbPDI11-2	GWHPAAYT044219	MALAVTSSSSASISGRTFTPSSEPKAPQLGSVRVFDRSYGQIQRQAVTFSQRRSSVRPVNAQPKRSESIVPSAATTAAPEVVEKVEVEDYAQLAKELENASPLEIMDKALEKFGNDIAIAFSGAEDVALIEYAHTGRPYRVFSLDTGRLNPETYQFFDTVEKHYGIRIEYMFPDAVEVQALVRTKGLFSFYEDGHQECCRVRKVRPLRRALKGLRAWITGQRKDQSPGTRSEIPVVQVDPVFEGLDGGAGSLVKWNPVANVADIWNFLRAMNVPVNSLHSQGYISIGCEPCTRSVLPGQHEREGRWWWEDAKAKECGLHKGNIKQEGGQNGMAAQSNDAATETDIFTSQNLVTLTRTGIENLAKLEDRKEPWIVVLYAPWCPFCQAMEASFVELADKLAGSSVKVGKFRADGEQKEFAQKELQLTSFPTILFFPKHSSQPIKYPTEKRDVDSLMAFVNALR
PbPDI11-3	GWHPAAYT036474	MAFVATSPSTQSLLHASLSSHEPKAQWTSSVNPSDQFSFASTAVASFQGPSLAKPLSAESKRSKSVVPLAAKMASPGIHVLRKLAKELENASPLQIMKKALDKFGNDIAIAFSGAEDVALIEYAKLTGRPFRVSLDTGRLNPETYQFFDTVEKHYGIHIEYMFPDAVEVQALVRGKGLFSFYEDGHQECCRVRKVRPLRRALKGLRAWITGQRKDQSPGTRAEIPVVQVDPSFEGIDGGIGSLVKWNPVANVEGHDIWNFLQMNVPVNPLHSQGYISIGCEPCTRPVLPGQHEREGRWWWEDAKAKECGLHNGNIKEKEALHLNGNGNGISTTNGSANVPDIFNSKNLVSLSRTGIENLARLENRSEPWLVVLYAPWCRFCQAMEGSYIELAEILAGTGVKVGKFRADGEQKKYAQQELKLGSFPTILFFPKHSSKPVKYPSENRDVASLLAFVNALR
ATPDI1-1	AT1G21750	MAMRGFTLFSILVLSLCASSIRSEETETKEFVLTLDHTNFTDTINKHDFIVVEFYAPWCGHCKQLAPEYEKAASALSSNVPPVVLAKIDASEETNREFATQYEVQGFPTIKIFRNGGKAVQEYNGPREAEGIVTYLKKQSGPASAEIKSADDASEVVSDKKVVVVGIFPKLSGSEFDSFMAIAEKLRSELDFAHTSDAKLLPRGESSVTGPVVRLFKPFDEQFVDSKDFDGEALEKFVKESSIPLITVFDKDPNNHPYVIKFFESTNTKAMLFINFTGEGAESLKSKYREVATSNKGQGLSFLLGDAENSQGAFQYFGLEESQVPLIIIQTADDKKYLKTNVEVDQIESWVKDFKDGKIAPHKKSQPIPAENNEPVKVVVSDSLDDIVLNSGKNVLLEFYAPWCGHCQKLAPILDEVAVSYQSDSSVVIAKLDATANDFPKDTFDVKGFPTIYFKSASGNVVVYEGDRTKEDFISFVDKNKDTVGEPKKEEETTEEVKDEL*

ATPDI1-2	AT1G77510	MAFKGFACFSILLLLSLFVSSIRSEETKEFVLTLDHSNFTETISKHDFIVVEFYAPWCGHCQK<br>LAPEYEKAASELSSHNPPLALAKIDASEEANKEFANEYKIQGFPTLKILRNGGKSVQDYNG<br>PREAEGIVTYLKKQSGPASVEIKSADSATEVVGEKNVVAVGVFPKLSGDEFDSFMALAEK<br>LRADYDFAHTLDAKFLPRGESVEGPAVRLFKPFDELFVDSKDFNGEALEKFVKESSIPLVT<br>VFDSDPNNHPYVAKFFESPATKAMMFVNFTGATAEALKSKYREVATSNKDQSLAFLVGD<br>AESSQGAFQYFGLEESQVPLIIIQTPDNKKYLKVNVEVDQIESWFKDFQDGKVAVHKKSQ<br>PIPAENNEPVKVVVAESLDDIVFKSGKNVLIEFYAPWCGHCQKLAPILDEVALSFQNDPSVI<br>IAKLDATANDIPSDTFDVKGFPTIYFRSASGNVVVYEGDRTKEDFINFVEKNSEKKPTSHG<br>EESTKSEEPKKTEETAAKDEL*
ATPDI2-1	AT3G54960	MASSSTSISLLLFVSFILLLVNSRAENASSGSDLDEELAFLAAEESKEQSHGGGSYHEEEHD<br>HQHRDFENYDDLEQGGGEFHHGDHGYEEEPLPPVDEKDVAVLTKDNFTEFVGNNSFAM<br>VEFYAPWCGACQALTPEYAAAATELKGLAALAKIDATEEGDLAQKYEIQGFPTVFLFVD<br>GEMRKTYEGERTKDGIVTWLKKKASPSIHNITTKEEAERVLSAEPKLVFGFLNSLVGSESE<br>ELAAASRLEDDLSFYQTASPDIAKLFEIETQVKRPALVLLKKEEEKLARFDGNFTKTAIAEF<br>VSANKVPLVINFTREGASLIFESSVKNQLILFAKANESEKHLPTLREVAKSFKGKFVFVYV<br>QMDNEDYGEAVSGFFGVTGAAPKVLVYTGNEDMRKFILDGELTVNNIKTLAEDFLADKL<br>KPFYKSDPLPENNDGDVKVIVGNNFDEIVLDESKDVLLEIYAPWCGHCQSFEPIYNKLGKY<br>LKGIDSLVVAKMDGTSNEHPRAKADGFPTILFFPGGNKSFDPIAVDVDRTVVELYKFLKK<br>HASIPFKLEKPATPEPVISTMKSDEKIEGDSSKDEL*
ATPDI2-2	AT5G60640	MAFRVLLLFSLTALLIFSAVSPSFAASSSDDVDDEDLSFLEDLKEDDVPGADSLSSSTGFDE<br>FEGGEEEDPDMYNDDDDEEGDFSDLGNPDSDPLPTPEIDEKDVVVIKERNFTDVIENNQY<br>VLVEFYAPWCGHCQSLAPEYAAAATELKEDGVVLAKIDATEENELAQEYRVQGFPTLLFF<br>VDGEHKPYTGGRTKETIVTWVKKKIGPGVYNLTTLDDAEKVLTSGNKVVLGYLNSLVGV<br>EHDQLNAASKAEDDVNFYQTVNPDVAKMFHLDPESKRPALVLVKKEEEKISHFDGEFVK<br>SALVSFVSANKLALVSVFTRETAPEIFESAIKKQLLLFVTKNESEKVLTEFQEAAKSFKGKL<br>IFVSVDLDNEDYGKPVAEYFGVSGNGPKLIGYTGNEDPKKYFFDGEIQSDKIKIFGEDFLN<br>DKLKPFYKSDPIPEKNDEDVKIVVGDNFDEIVLDDSKDVLLEVYAPWCGHCQALEPMYN<br>KLAKHLRSIDSLVITKMDGTTNEHPKAKAEGFPTILFFPAGNKTSEPITVDTDRTVVAFYKF<br>LRKHATIPFKLEKPASTESPKTAESTPKVETTETKESPDSTTKSSQSDSKDEL*
ATPDI3-1	AT1G52260	MSLIPKPISKVSTFTFILLILLSFTIIIAYSSPDSNVESNEPGFDSDLDQLLAVDEQLQEDRPEQ<br>QSEAETVSKAQRIVLELNGDYTKRVIDGNEFVMVLGYAPWCARSAELMPRFAEAATALK<br>EIGSSVLMAKIDGDRYSKIASELEIKGFPTLLLFVNGTSLTYNGGSSAEDIVIWVQKKTGAP<br>IITLNTVDEAPRFLDKYHTFVLGLFEKFEGSEHNEFVKAAKSDDEIQFIETRDSDVAKLLFP<br>DLKSNNVFIGLVKPEAERYTVYDGSYKMEKILEFLGSNKFPLFTKLTETNTVWVYSSPVK<br>LQVMLFSKADDFQKLAQPLEDIARKFKSKLMFIYVDITNENLAMPFLILFGIEAGNKTVVA<br>AFDNNLNSKYLLESDPSPNSIEEFCSGLAHGTVSRYYRSEPVPDNENASIVTVVGKTFDGL<br>VLNSRENVLLEVHTPWCVNCEALSKQIEKLAKHFKGFENLVFARIDASANEHTKLQVDD<br>KYPIILLYKSGEKEKPLKLSTKLSAKDIAVFINEELLKPKNGSAKDEL*

ATPDI3-2	AT3G16110	MLTKPKPNSKFSILFTFLLLLSFLIFVARSSDVAVEAGSEEELDDLEQLLAVDEQLQEERPE<br>QQSEAETVSKAQRIVVELNGDNTKRLIDGNEYVMVLGYAPWCARSAELMPRFAEAATDL<br>KEIGSSVLMAKIDGERYSKVASQLEIKGFPTLLLFVNGTSQSYTGGFSSEEIVIWVQKKTGA<br>STIKLDTVDEASGFLKKHHTFILGLFEKSEDSSGHDEFVKAASLDNEIQFVETSSIDVAKLL<br>FPNLKTNNVFVGLVKTEAEKYTSYDGPCQAEKIVEFLNSNKFPLVTKLTESNTVRVYSSPV<br>KLQVMVFSKTDDFESLAQPLEDIARKFKSKLMLIYIDISNENLAMPFLTLFGIEDAKKTVV<br>AAFDNNLNSKYLLESDPSPSNIEEFCFGLAHGTVSAYYKSQPIPDNQNASVVAVVGRTFDE<br>VVLRSSENVLLEVHTPWCINCEALSKQVEKLSQHFKGFENLVFARIDASANEHPKLTVDD<br>YPTILLYKTGEKENPLKLSTKSSAKDMAVLINKELKWKDQSGKDEL*
ATPDI4-1	AT2G47470	MAKSQIWFGFALLALLLVSAVADDVVVLTDDSFEKEVGKDKGALVEFYAPWCGHCKKL<br>APEYEKLGASFKKAKSVLIAKVDCDEQKSVCTKYGVSGYPTIQWFPKGSLEPQKYEGPRN<br>AEALAEYVNKEGGTNVKLAAVPQNVVVLTPDNFDEIVLDQNKDVLVEFYAPWCGHCKS<br>LAPTYEKVATVFKQEEGVVIANLDADAHKALGEKYGVSGFPTLKFFPKDNKAGHDYDGG<br>RDLDDFVSFINEKSGTSRDSKGQLTSKAGIVESLDALVKELVAASEDEKKAVLSRIEEEAS<br>TLKGSTTRYGKLYLKLAKSYIEKGSDYASKETERLGRVLGKSISPVKADELTLKRNILTTF<br>VASS*
ATPDI5-1	AT1G04980	MERKMYKSTVFPICCLLFALFDRGNALYGSSSPVLQLTPSNFKSKVLNSNGVVLVEFFAP<br>WCGHCQSLTPTWEKVASTLKGIATVAAIDADAHKSVSQDYGVRGFPTIKVFVPGKPPIDY<br>QGARDAKSISQFAIKQIKALLKDRLDGKTSGTKNGGGSSEKKKSEPSASVELNSSNFDELV<br>TESKELWIVEFFAPWCGHCKKLAPEWKKAANNLKGKVKLGHVNCDAEQSIKSRFKVQGF<br>PTILVFGSDKSSPVPYEGARSASAIESFALEQLESNAGPAEVTELTGPDVMEDKCGSAAICF<br>VSFLPDILDSKAEGRNKYLEMLLSVADKFKKDPYGFVWVAAGKQPDLEKRVGVGGYGY<br>PAMVALNAKKGAYAPLKSGFEVKHLKDFVKEAAKGGKGNLPIDGTMEIVKTEAWDGKD<br>GEVVDADEFSLEDLMGNDDEASTESKDDL*
ATPDI5-2	AT2G32920	MYKSPLTLLTLLTICFGFFDLSSALYGSSSPVVQLTASNFKSKVLNSNGVVLVEFFAPWCG<br>HCKALTPTWEKVANILKGVATVAAIDADAHQSAAQDYGIKGFPTIKVFVPGKAPIDYQGA<br>RDAKSIANFAYKQIKGLLSDRLEGKSKPTGGGSKEKKSEPSASVELNASNFDDLVIESNEL<br>WIVEFFAPWCGHCKKLAPEWKRAAKNLQGKVKLGHVNCDVEQSIMSRFKVQGFPTILVF<br>GPDKSSPYPYEGARSASAIESFASELVESSAGPVEVTELTGPDVMEKKCGSAAICFISFLPDI<br>LDSKAEGRNKYLEMLLSVAEKFKKQPYSFMWVAAVTQMDLEKRVNVGGYGYPAMVA<br>MNVKKGVYAPLKSAFELQHLLEFVKDAGTGGKGNVPMNGTPEIVKTKEWDGKDGELIE<br>EDEFSLDELMGGDDAVGSKDEL*
ATPDI6-1	AT1G07960	MTLGARLVAPMIILLLFIPIELVKAEVITLTPETFSDKIKEKDTAWFVKFCVPWCKHCKKLG<br>NLWEDLGKAMEGDDEIEVGEVDCGTSRAVCTKVEIHSYPTFMLFYNGEEVSKYKGKRDV<br>ESLKAFVVEETEKAAEKAQLEDKEL*
ATPDI7-1	AT1G35620	MRSLKLLLCWISFLTLSISISASSDDQFTLDGTVLELTDSNFDSAISTFDCIFVDFYAPWCGH<br>CKRLNPELDAAAPILAKLKQPIVIAKLNADKYSRLARKIEIDAFPTLMLYNHGVPMEYYGP<br>RKADLLVRYLKKFVAPDVAVLESDSTVKEFVEDAGTFFPVFIGFGLNESIISGLGRKYKKK<br>AWFAVSKEVSEDTMVSYDFDKAPALVANHPTYNEHSVFYGPFEDGFLEEFVKQSFLPLIL<br>PINHDTLKLLKDDERKIVLTIVEDETHESLEKLYKALRAAAHANRDLVFGYVGVKQFEEF<br>VDSFHVDKKTNLPKIVVWDGDEEYDQVTGIETITQEEDHLTQVSRFLEGYREGRTEKKKI<br>NGPSFMGFINSMIGIRSVYILVFLVAVIMMLRSLGQVEEPTGVRTATAVRERVDQATTVPE<br>DESSEHKPSDKKED*

ATPDI8-1	AT3G20560	MVSSTKLKSVDFYRKIPRDLTEASLSGAGLSIVAALFMMFLFGMELSSYLEVNTTTAVIVD<br>KSSDGDFLRIDFNISFPALSCEFASVDVSDVLGTNRLNITKTVRKFPIDPHLRSTGAEFHSGL<br>ALHNINHGEETKEEFPDGAIPLTSASFEALSHHFPILVVNFNAPWCYWSNRLKPSWEKAAN<br>IIKQRYDPEADGRVLLGNVDCTEEPALCKRNHIQGYPSIRIFRKGSDLREDHGHHEHESYY<br>GDRDTDSIVKMVEGLVAPIHPETHKVALDGKSNDTVKHLKKGPVTGGCRVEGYVRVKK<br>VPGNLVISAHSGAHSFDSSQMNMSHVVSHFSFGRMISPRLLTDMKRLLPYLGLSHDRLDG<br>KAFINQHEFGANVTIEHYLQTVKTEVITRRSGQEHSLIEEYEYTAHSSVAQTYYLPVAKFH<br>FELSPMQILITENPKSFSHFITNLCAIIGGVFTVAGILDSIFHNTVRLVKKVELGKNI*
ATPDI8-2	AT4G27080	MVSTSKIKSVDFYSDPEVVGFASRVCALSLSFDLSDIELFRCYFLGFKMIVSHFDFGIVAKR<br>VASKKIPRDLTEASLSGAGLSIIAALSMIFLFGMELNNYLAVSTSTSVIVDRSADGDFLRLD<br>FNISFPSLSCEFASVDVSDVLGTNRLNVTKTIRKFSIDSNMRPTGSEFHAGEVLSLINHGDET<br>GEEIVEDSVPLTGRNFDTFTHQFPILVVNFYAPWCYWCNLLKPSWEKAAKQIKERYDPEM<br>DGRVILAKVDCTQEGDLCRRNHIQGYPSIRIFRKGSDLKDDNAHHDHESYYGDRDTESLV<br>KMVVSLVEPIHLEPHNLALEDKSDNSSRTLKKAPSTGGCRVEGYMRVKKVPGNLMVSAR<br>SGSHSFDSSQMNMSHVVNHLSFGRRIMPQKFSEFKRLSPYLGLSHDRLDGRSFINQRDLGP<br>NVTIEHYLQIVKTEVVKSNGQALVEAYEYTAHSSVAHSYYLPVAKFHFELSPMQVLITEN<br>SKSFSHFITNVCAIIGGVFTVAGILDSILHHSMTLMKKIELGKNF*
ATPDI8-3	AT1G50950	MVSTSKIKSVDFYRKIPRDLTEASLSGAGLSIVAALAMLFLFGMELSSYLAINTSTSVIVDK<br>SSDGDFLNIDFNISFPALSCEFASVDVSDVFGTHRLNISKTIRKVPIDPHLRATAEEFHSTSD<br>LHLINHGDEDHGDNSTYADIPLTGAAFEKFTHHFQILVVNFYAPWCYWSNRLKPSWVKA<br>SQITRERYNPGTDDRVLLGSVDCTEEPTLCKSLPIFSNHIQGYPSIRIFRRGSGLREDHGNHE<br>HESYYGDRDTDSLVKMVEELLKPIKKEDHKLALDGKSDNAASTFKKAPVSGGCRIEGYV<br>RAKKVPGELVISAHSGAHSFDASQMNMSHIVTHLTFGTMVSERLWTDMKRLLPYLGQSY<br>DRLNGKSFINERQLDANVTIEHYLQIIKTEVISRRSGQEHSLIEEYEYTAHSSVARSYHYPE<br>AKFHFELSPMQVLISENPKSFSHFITNVCAIIGGVFTVAGILDSIFQNTVRMVKKIELGKNI*
ATPDI9-1	AT1G15020	MSLIHLFLLLGLLSLEAAASFSPGSRSILRDIGSNVADQKDNAIELNATNFDSVFQDSPAKY<br>AVLEFFAHWCPACRNYKPHYEKVARLFNGADAVYPGVVLMTRVDCAIKMNVKLCDKFS<br>INHYPMLFWAPPKRFVGGSWGPKQEKNEISVVNEWRTADLLLNWINKQIGSSYGLDDQK<br>LGNLLSNISDQEQISQAIFDIEEATEEAFDIILAHKAIKSSETSASFIRFLQLLVAHHPSRRCRT<br>GSAEILVNFDDICPSGECSYDQESGAKDSLRNFHICGKDVPRGYYRFCRGSKNETRGFSCG<br>LWVLMHSLSVRIEDGESQFAFTAICDFINNFFMCDDCRRHFHDMCLSVKTPFKKARDIAL<br>WLWSTHNKVNERLKKDEDSLGTGDPKFPKMIWPPKQLCPSCYLSSTEKNIDWDHDQVYK<br>FLKKYYGQKLVSVYKKNGESVSKEEVIAAAEEMAVPTNALVVPVGAALAIALASCAFGA<br>LACYWRTQQKNRKYNYNPHYLKRYNSNYMVMNTFSNTESEREKER*
ATPDI9-2	AT2G01270	MSLVHLLLFAGLVIAASSSSPGSRLILREISDQKDKAVELNTTNFDSVLKDTPAKYAVVEFF<br>AHWCPACRNYKPHYEKVARLFNGPDAIHPGIVLMTRVDCAMKTNTKLCDKFSVSHYPM<br>LFWGPPTKFVSGSWEPKKDKSEILVIDDGRTAERLLNWINKQIGSSYGLDDQKFKNEHAL<br>SNLTDYNQISQAVYDVEEATAEAFDIILAHKAIKSSETSASFIRFIQLLAAHHLSRRCRKGA<br>AEILVNYDDLCPSGNCSYEKSGGNDTLGNFPICGKDVPRGYYMFCRGSKNDTRGFSCGL<br>WVLMHSLSVRIEDGESHFAFTTICDFVNNFFMCDECRLHFNDMCLSVKTPFKKARDFVL<br>WVWSTHNKVNERLLKDEASLGTGDPKFPKIIWPPKELCPLCYLSSNQKSIEWDHEHVYKF<br>LKNYYGPKLVSLYKEKSVSRSKEETVSATEDLTVATNALVVPIGAALAIAIASCAFGALAC<br>YWRTQQKNRKPRRR*

ATPDI10-1	AT3G03860	MDSRVSILFVCAIAVSCFTSGSASSPVDFSVCNYEFELFRFDLEAKCPPSLYPTPPIEVDGDS<br>LDRLMASQHGNAYMSVLFYASWCPFSRAVRPKFDMLSSMFPQIQHLAVEHSQALPSVFS<br>RYGIHSLPSILMVNQTLNARYHGRKDLISLIEFYEEATGLQPVQYVAEGEPTGLNAGDGNL<br>ITWLRKGTSIREIFKQDPFLVLSLLFICLQMAILVFPIAESRMRALWASYVANLNLGRFGEIS<br>QLFNRGIHMVDVRRLWLKLSLVKTRNFHERAKNAQAWASSLASVSLGQTSSDQS*
ATPDI10-2	AT1G34780	MEKEILLLLLVIMFLTVADVDAVRVPFCATKSAKDSIFGLRDQTCSVSGVESDERPRFVAV<br>TEGDERWLQIALDMIHKNKCDYVALLFYASWCPFSRSFRPSFDVISSLYSSIPHFAIKESSIK<br>PSTLSKYGVHGFPTLLLLNSTMRARYRGTRMLDSLVAFYSDVTGIETLDKTSLERSVSVPH<br>LGNENNTEPENCPFTWARSPENMLRQETYLALAIVFVLLRLLHLIYPTLVVFMKFTWRRIA<br>QNMRLESLLEHTVGFLSRAVQLCMHRRSNLQGGAMNARAWASKSLATVSIGDSSSSNRR<br>SSSSQ*
ATPDI11-1	AT4G21990	MALAINVSSSSSSAISSSSFPSSDLKVTKIGSLRLLNRTNVSAASLSLSGKRSSVKALNVQSI<br>TKESIVASEVTEKLDVVEVEDFEELAKRLENASPLEIMDKALEKFGNDIAIAFSGAEDVALI<br>EYAHLTGRPYRVFSLDTGRLNPETYRLFDTVEKHYGIRIEYMFPDAVEVQALVRNKGLFS<br>FYEDGHQECCRIRKVRPLRRALKGLRAWITGQRKDQSPGTRSEIPVVQVDPVFEGLDGGV<br>GSLVKWNPVANVEGNDVWNFLRTMDVPVNTLHAAGYVSIGCEPCTRAVLPGQHEREGR<br>WWWEDAKAKECGLHKGNIKENTNGNATANVNGTASVADIFNSENVVNLSRQGIENLMK<br>LENRKEAWIVVLYAPWCPFCQAMEASFDELADKLGGSGVKVAKFRADGDQKDFAKKEL<br>QLGSFPTILVFPKNSSRPIKYPSEKRDVDSLTSFLNLVR*
ATODI11-2	AT1G62180	MALAVTSSSTAISGSSFSRSGASSESKALQICSIRLSDRTHLSQRRYSMKPLNAESHSRSES<br>WVTRASTLIAPEVEEKGGEVEDFEQLAKKLEDASPLEIMDKALERFGDQIAIAFSGAEDVA<br>LIEYARLTGKPFRVFSLDTGRLNPETYRLFDAVEKQYGIRIEYMFPDAVEVQALVRNKGLF<br>SFYEDGHQECCRVRKVRPLRRALKGLKAWITGQRKDQSPGTRSEIPIVQVDPVFEGLDGG<br>VGSLVKWNPLANVEGADVWNFLRTMDVPVNALHAQGYVSIGCEPCTRPVLPGQHEREG<br>RWWWEDAKAKECGLHKGNIKEEDGAADSKPAAVQEIFESNNVVALSKGGVENLLKLEN<br>RKEAWLVVLYAPWCPFCQAMEASYIELAEKLAGKGVKVAKFRADGEQKEFAKQELQLG<br>SFPTILLFPKRAPRAIKYPSEHRDVDSLMSFVNLLR*
ATPDI11-3	AT4G04610	MAMSVNVSSSSSSGIINSRFGVSLEPKVSQIGSLRLLDRVHVAPVSLNLSGKRSSSVKPLNA<br>EPKTKDSMIPLAATMVAEIAEEVEVVEIEDFEELAKKLENASPLEIMDKALEKYGNDIAIAF<br>SGAEDVALIEYAHLTGRPFRVFSLDTGRLNPETYRFFDAVEKHYGIRIEYMFPDSVEVQGL<br>VRSKGLFSFYEDGHQECCRVRKVRPLRRALKGLKAWITGQRKDQSPGTRSEIPVVQVDPV<br>FEGLDGGVGSLVKWNPVANVEGNDVWNFLRTMDVPVNTLHAAGYISIGCEPCTKAVLP<br>GQHEREGRWWWEDAKAKECGLHKGNVKENSDDAKVNGESKSAVADIFKSENLVTLSR<br>QGIENLMKLENRKEPWIVVLYAPWCPFCQAMEASYDELADKLAGSGIKVAKFRADGDQ<br>KEFAKQELQLGSFPTILVFPKNSSRPIKYPSEKRDVESLTSFLNLVR*
BrPDI1-1	Bra016405	MAMRGYALFSILALSLLASSVRSEETATETTKEFVLTLDHTNFTDTVNKHDFIVVEFYAPW<br>CGHCKQLAPEYEKAASELSSHVPPVVLAKIDASEETNREFATQYEVQGFPTIKIFRNGGKA<br>VQEYNGPREADGIVTYLKKQSGPASFEIKAAEDASEFDKKVIVVGVFPKLSGSEFDSFLAT<br>AEKLRSDYDFAHTSDAKLLPRGESVTGPVVRLFKPFDELFVDSKDFDGEALEKFVKESSIP<br>LITVFDKDPNNHPYVIKFFDSSNTKAMLFINFTGEGAESLKSKYREVATSYKGQGLSFLLG<br>DAENSQGAFQYFGLEESQVPLIIIQTVDDKKYLKTNIEIDQIESWVKDFKDGKVAPHKKSQ<br>PIPTENNEPVKVVVAESLDEMVFNSGKNVLLEFYAPWCGHCQKLVPILDEVAVSYQSDPS<br>VVIAKLDATANDFPNDTFDVKGFPTIYLRSASGNIVLYDGDRTKEDIISFIDKNKDTAGEPK<br>KEETTTEAVKDEL

BrPDI1-2	Bra012293	MAAMRRGYALFSILALSLLASSVRSETKEFVLTLDHSNFTDTINKHDFIVVEFYAPWCGHC<br>KQLAPEYEKAASELSSNVPAVVLAKIDASEETNKEFATKYEVQGFPTIKIFRNGGKAVQEY<br>KGPREADGIVS<br>YLKKQSGPASFEIKSGDDVVGDKKVVVVGVFPKLAGSEFDSFLATAEKLRSDYDFAHTSD<br>AKLLPRGESVTGPVVRLFKPFDELFVDSKDFDGEALEKFVKESSIPLITVFDKDPNNHPYVI<br>KFFDSPNTKAMF<br>FINFTGESAETLKSKYREVATSNKGQGLSFLLGDAENSQGAFQYFGLEESQVPLIIIQTADD<br>KKYLKTNVEVDQIGSWIKDFKDGKVSPHKKSQPIPTENNEPVKVVVGESLDDMVFNSGK<br>NVLLEFYAPWC<br>GHCQKLVPILVEVAVSYQSDPSVVIAKLDATANDFPRDTFDVKGFPTIYFRSASGNVVLYE<br>GDRTKEDFISFIDKNKDTAGEPKTEDKTAEATKDEL
BrPDI1-3	Bra017948	MAMKGYTLCSILVFSLFASCVRSKETKEFVLTLDHTNFTETINKHDFIVVEFYAPWCGHCK<br>QLAPEYEKAASELSSHVPPVVLAKIDASEETNKEFATKYSVQGFPTIKILRNGGKAVQEYN<br>GPREADGIVTYLKKQSGPASLEIKSADAASEVVGDKNVVAVGVFPKLSGAEFDSFMATAE<br>KLRSDYDFAHTTDAKLLPRGESVTGPVVRLFKPFDELFVDFRDFVGEALEKFVKESSIPLIT<br>VFDSDPNNHPYVLKFFEIPNTK<br>ALFFLNFNGEGAETLKSKYREVAASNKGHGLSFLLGDAKNSEEALQHYGVEQRQLPLIIL<br>QTVDDKKYLKTNVEVDQIESWINDFKDGKASPYKKSQPIPGENNEPVKVVVAENLDEMV<br>FSSGKNVLLEFY<br>APWCGHCQNLVPILDEVAVSYQSDPSVVIAKFDATANDFPHDTFDVKGFPTIYLRSANGNI<br>VLYKGDRTKEDIISFIDKNKDTAGETKTEEKKTKEVKDEL
BrPDI1-4	Bra008311	MAFKGFALFSIVVLSIFASSRSEETETKEFVLTLDHSNFTETINKHDFIVVEFYAPWCGHCK<br>SLAPEYEKAASELITHNPPLVLAKIDASEESNKGIANEYKIQGFPTIKILRNGGKSIQDYNGP<br>REAPGIVSYVKK<br>QSGPASSEIKTAADAAEVVGEKNVVAVGVFPKLSGEEFDSFIALAEKLRGDYDFAHTLDA<br>KLLPRGDSSVAGPVVRLFKPFDELFVDSKDFNGEALEKFLKESSIPLVTVFDSDPSNRPYV<br>ASFFDSSATKVM<br>MFVNFTGESAESLKSKFRKVATSYKGQDLSFLVGDAEGGKGALEYFGVEESQVPLVIIQTP<br>DSKKYLKANVVVEEIESWMKDFKDGKVDVFKKSQPIPAENNEPVKVVVAETLDDIVLKS<br>GKNVLIEFYAP<br>WCGHCQKIAPILDEVALAFKNDPSVIIAKLDATANDIPSEPFDVKGFPTIYFRSVSGTVVAY<br>EGNRTKEDFISFIEKNKPTTSHVEDTTSSTKTEEPKKIDDASDTKDEL
BrPDI1-5	Bra015665	MASNGFAMLSILVLALFASSIRSEETETKEFVLTLDHSNFTDTINKHDFIVVEFYAPWCGHC<br>KSLAPEYEKAAAELSSQSPPIFLAKIDASEESNKGIANDYKIQGFPTIKILRKGGKSIQDYNG<br>PREAAGIVTYV<br>KKQSGPASAEIKSADGAGEVIGEKSVVAVGVFPKLSGEEFDSFMALAEKLRADYDFAHTL<br>DAKLLPRGDSSVAGPVVRLFKPFDELFVDSKDFNGEALEKFVKESSIPLVTVFDKDPSNHP<br>YVSKFFDNPATK<br>VMMFVNFTGETAESLKSKFREVATSSKGQDLAFLVGDAESSQGALQYFGLEESQVPLIIIQ<br>TPDSKKYLKANVVVDQIESWMKDFKDGKVAAHKKSQPIPAENNEPVKVVVAESLDEMV<br>FNSGKNVLIEFY<br>APWCGHCQKLAPILDEVALAFQNDPSVIVAKLDATANDIPSDTFDVKGFPTIYFRSADGKV<br>VVYEGSRTKEDFISFIEKNKPASHSEESSTTVRSGEHKTEESAAKDEL

BrPDI2-1	Bra007120	MASSTSMSLLFLLSFLLLATSRAENAANGSDLDEELAFLAAEESKEEQQHHANSHHDQYR<br>DFENYEDLEQGGEFHHGEHEGGGEYHEEEPQLPIVDEKDVAVLTKDNFTEFVGNNSFAM<br>VEFYAPWCGAC<br>QALAPEYAAAATELKGVAALAKIDATEEGDLAQKYEIQGFPTVFLFVDGEMRKTYEGER<br>TKDGIVTWMKKKASPSIHNITTVEEAERVLSAEPKVVLAFLDSLVGSESAELAAASRLEDD<br>LSFYQTTSPDIA<br>KLFEIETEVKRPALVLLKKEEEKLARFDGNFTKAAISEFVSANKSPLVINFTREGASLIFENS<br>VKNQLILFATTNESEKHLPTLREVAKSFKGKFVFVYVQMDNEDYGEAVSGFFGVTGTAPK<br>VLVYTGNEDM<br>RKFILDGELTVNNIKTLAEDFLADKLKPFYKSDPVPETNDGDVKIIVGNNFDEIVLDESKD<br>VLLEIYAPWCGYCQSFEPIYNKLGKYLKGIDSLVVAKMDGTTNEHPRAKADGFPTILFFPG<br>GNKSFDPITVDV<br>DRTVVELYKFLKKHASVPFKLAKPSATPEQVITTKKADEKTESDGAKDEL
BrPDI2-2	Bra002464	MAFRVFLLLSLTALLIFSAVSPSFSTSDVDDEDLSFLEDPKEEHDPTKPLTSTESELDEFNEG<br>EEEDPEMYEGDDEEEGEDLSDLGNPDSDPFPTPDVDEKDVVVVKERNFTDVIENNQYVM<br>VEFYAPWCGHC<br>QSLAPEYAAAATELKGDGVVLAKIDATEENELAHQYSVQGFPTILFFVDGEHKPYTGGRT<br>KDTIVTWVKKKIGPSVYNLTTLDDAEKVLTSGNKVVLGYLNSLVGVEHDQLAAASKAED<br>DVNFYQTVNPD<br>VAKLFHIDPEAKRPAVVLVKREAEKISHFDGEFVKSDLASFVSANKLPLVSVFTRESAPEIF<br>ESAIKKQILLFVTQNGSEKVLPEFEEAAKSFKGKLIFVSVDLDNEDYGKPVAEYFGVSGNG<br>PKLIAYTGNEDP<br>KKHFFDGEIKSDKIKTFAEEFLSDKLKPFYKSDPIPEKNDGDVKIVVGDNFDDIVLDESKDV<br>LLEVYAPWCGHCQALEPMYNKLAKHLREIDSLVIAKMDGTTNEHPKAKAEGFPTILFFPA<br>GNKTAEPITVD<br>TDRTVVAFYKFLRKHATIPFKLEKPAASTESPKTAKSTPKVETTETKGNPQSTTKSTESDL<br>KDEL
BrPDI2-3	Bra020239	MASRVFLLLSLTALLIFSAVSPSLSADVDDEEDLSFLEDLTEEVKAPAKPLTDDFEGGEDD<br>DDEEEDGEHFSDVSNQDSDPFPLSDVDEKDVVVVKERNFTDVIENNEYVMVEFYAPWRG<br>HCQSLAPEYAA<br>AATELKGDGVVLAKIDATVENELAHQYSVQGFPTILFFVDGEHKLYTGGRTKETIVTWVK<br>KKIGPSVYNLTTLDDAEKVLTSGNKVVLGYLNSLVGVEHDQLAAASKAEDDVNFYQTVN<br>PDVAKMFHIDP<br>ESKRPALVLVKREEEKISHFDGEFVKSGLVSFVSANKLPLVTVFTPESSQEIFESAIKKQLLL<br>FATENGSEKVLQEFEEAATLFKGKLIFVSVDVDNEDYGKPVAEYFGVSSSNAPKLVAFTG<br>NEDPQKHYFEG<br>EIKSDKIKIFGEEFLSDKLKPFYKSDPIPEKNDGDVKIVVGDNFDEIVLDESKDVLLEVYAP<br>WCGHCQALEPMYNKLAKHLRSIDSVVIAKMDGTTNEHPKAKAEGFPTVLFFPAGNKTSS<br>EPITVDADRTVV<br>AFYKFLRKHATIPFKLEKPAASTESPTAAESTPKVETTETKGKLESTTTKSTESDSKDEL

BrPDI3-1	Bra014319	MSMNPKLSVSTFILLLLLTFLLIPSHSSSDEESDDDLEQLLAVDEQSQEDRPQHQQSEAET<br>VSKAQRIVLELTGDNAKRVVDGNEFVLVLGYAPWCARSADLMPRFSEAATGLKEIGSSV<br>LMAKIDGDRYGKVASELEIKGFPTLLLFVNGTSQPYSGGFSAEDIVIWVQKKTGSPIITVNT<br>LDEAQIFLNKYHTFVVGLFHKFEGSEYNEFVKAAKSDNEIQFVETSDNDVAKLLFPQLKT<br>NTVFIGLVKPEAERYTAYDGPFKM<br>EKLLEFLGNNKFPLITRLTESNTVWVYSSPVKLQVMLFSKAYVFQSLAQPLEDLARKFKS<br>KLMFIYVDIANENLAMPFLTLFGIEHANKTVDFCSGLADGTVSRYYRSEPVPDNVDDFPTI<br>LLYKSGEKEKPV<br>ITFLIEQSYKPSM
BrPDI3-2	Bra018958	MSINPKPQSSLLTFILLLLLTSAAYSSSNHPGSDEESDDLEQLLAVDEQLQQDLPLHHQQSE<br>AETVSRAQRIVLELSGDNARRVVGGNEFVMVLGYAPWCARSADLMPKFSEAATALKEIG<br>SPVVMAKIDGDRYGKVASEMEIKGFPTLLLFVNGTSKAYTGGFSAEEIVIWVQKKTGAPI<br>VTVNTVDEAQRFLKKYHTFVVGLFNKFEGSEYNEFVKAAKSDDEIQFVETSDSEVAKLLF<br>PEIKTSDVFIGMVKTEAERYTSYA<br>GSYKMENILEFLSKNKFPLITKLSESNTAWVYSSPVKLQVMIFAKADDFQNMAQPLENFA<br>RRFKSKLMFIYIDITNENLAMPFLTLFGIEHANKTVVAAFDNKLNSKYLLESDPSPTNIEDF<br>CSGLADGTIPQY<br>YRSEPVPDNENASIVTVVGKTFDELVLNSQENVLLEVHTPWCVNCEAMSKQVVKLAKHF<br>KGFENLVFARIDASTNEHAKLQVNDYPTILLYKSGEKEKPLKISTKLSAKDMAVFINEELK<br>PRGGSAKDEL
BrPDI4-1	Bra000454	MAKSQIWFGLASLVALLVVSAVADDVVVLTDDSFEKEVGKDRGALVEFYAPWCGHCKK<br>LAPEYEKLGASFKKAKSILIAKVDCDEHKSVCTKYGVSGYPTIQWFPKGSLEPQKYEGAR<br>NAEALAEYVNK<br>EGGTNVKLAAAPQNVVVLTPDNFDEIVLDQNKDVLVEFYAPWCGHCKSLAPVYEKVAT<br>VFKQEEGVVIANLDADAHKSLGEKYGVSGFPTLKFFPKDNKAGQDYEGGRDLDDFVGFI<br>NEKVGTSRDSQG<br>QLTSKAGIVESLDALVKELVAASEDEKKTILSRIEEEASNLKGSTTRYGKLYSKLAKSYIEK<br>GSAYATKEVERLGRVLGKSISPVKADELTLKKNILSTFVASS
BrPDI4-2	Bra004455	MAKSQIWFGLALVALLVVSAVADDVVVLTEDSFEKEVGKDKGALVEFYAPWCGHCKKL<br>APEYEKLAASFKKAKSVLIAKVDCDEHKGVCTKYDVSGYPTIKWFPKGSLEPQKYEGPRN<br>AEALAEFVNKE<br>GGTNVKLAAVPQNVVVLTPDNFDEIVLDQNKDVLVEFYAPWCGHCKSLAPVYEKVATV<br>FKQEDGVVIANLDADAHKSLGEKYGVSGFPTLKFFPKDNKAGQDYDGGRDLDDFVTFIN<br>EKVGTSRDSKGQLTSKAGVVESLDALVKELVAASEDEKKAILSRIEEEASNLKGSTARYG<br>KLYSSLAKKYIEKGSGYATKEAERLGRVLSKSMSPVKADELTLKRNILNTFVASS
BrPDI5-1	Bra015375	MNKTRVFTILSLVFAFSFDLSNALYGSSSPVLQLTPSNFKSKVINSNGVVLVEFFAPWCGH<br>CKSLTPTWEKVATTLKGIATVAAIDADAHKSVSQDYGVRGFPTIKVFVPGKPPIDYQGAR<br>DAKAISQFAIKQI<br>KALLKDRLDGKTTGTTTGGGSSEKKSEPSASVELNSSNFDELVTESKDLWIVEFFAPWCG<br>HCKKLAPEWKKAAKNLKGKVKLGHVDCDADKAIQSRFKVKGFPTILVFGADKSSPLPYE<br>GARSASAIESFAL<br>EQLEANAGPAEVTELTGPDAMEEKCGPAAICFVSFLPDILDSKAEGRNKYLEMLLSVAEK<br>FKKDPISFVWVAAGKQPDLEKRVGVGGYGYPAMVALNAKKGAYAPLKSGFEVKHLIEF<br>VKEAQKGGKGNL<br>PIDGTLEIVKTEAWDGKDGEVVDADEFSLEELMADD

BrPDI5-2	Bra005546	MQNKSPLTLLTLLCLSLGFLNLTNALYGSSSPVVQLTASNFKSKVLNSNGVVLVEFFAPW<br>CGHCKALTPTWEKVASVLKGVATVAAIDADAHQSAAQDYGIQGFPTIKVFVPGKPPVDY<br>QGARDAKSIANF<br>AYKQIKALLSDRLEGKSKPSGGGSSEKKSEPSASVELNSSNFDELVIKSNDLWIVEFFAPW<br>CGHCKKLAPEWKRAAKNLKGKVKLGHVNCDVEQSIMSRFKVQGFPTIMVFGVDKSSPY<br>AYDGARSASAIESFATELVEASAGPVEVTELTGPDVMEKKCGSAAICFVSFLPDILDSKAE<br>GRNKYLEMLLSVAEKFKRHPYSFVWVAAVTQPDLEKRVNVGGYGYPAMVAMNVKKGV<br>YAPLKSAFELQHLLEFVKDAGAG<br>GKGNVPMNGTPEIVETKAWDGKDGEVMEEDEFSLEELMGGDDDANVGTKDEL
BrPDI6-1	Bra018672	MKLGARLIAFILLLSLTIVLTKAEVITLTPETFSDKVKEKDTAWFVKFCVPWCKHCKKLGN<br>LWEELGNAMEGDDEIEIGEVDCGKSRDVCTKVEIHSYPTFKLFYNGEEVSKYQGKRDVES<br>LKTFVVEETEK<br>AAEKAQLEDKEL
BrPDI7-1	Bra034408	MRSLGLMYWWISFLALSISLSASSDDQFTIDGTVLELTDSNFESAISTFDCVFVDFYAPWC<br>GHCKRLNPELDAAAPILAKLKQPIIIAKLNADKYSRLARKLEIDAFPTLMLYNHGVPMEYY<br>GPRKADLLVRY<br>LKKFVAPDVAVLESNSHVKDFVEDSGTSFPVFIGFGLNQSLISGLGRKYKKKAWFAVAKD<br>ASEDVMVSYDFDKAPALVAQHPAYNEHSVFYGPFEDGFLEEFVKQNFLPLILPINHDTLKL<br>LKDDERKMVL<br>TIVEDETHESMGKLIKALRAAAHANRDLVFGYVGVEQFEEFADSFHADKKAKLPKIVVW<br>DGDEEYEQVNGIETVSHEEDHLTQVSRFLEGYREGKTEKKRIKGPSFMGFINSMIGIRSVYI<br>IVFLVAVIMMLR<br>SLGQVEEPARVRTAASDGQATSVLEGETSEHKPRDKED
BrPDI8-1	Bra001793	MVSPTKLKSVDFYRKIPRDLTEASLSGAGLSIVAALVMMLLFGMELSSYLEVTTTTAVVV<br>DKSSDGDFLRIDFNISFPALSCEFASLDVNDVLGTNRLNITKTVRKFPIDPHLKATGGEFHS<br>GLASHHINHGEEI<br>KQEFPDGAIQLTNGGFQSLSHHFPLLIVNFNAPWCYWSNRLKPSWEKAATIIKQRYNPDT<br>DGRVLLGSVDCTEEPALCRRNHIQGYPSIRIFRKGNDLKEDHGHHEHESYYGDRDTESIVK<br>MVDELVAPIHPETHKLALDWGISNDTAKLLKKAPVTGGCRVEGYVRVKKVPGNLVISAH<br>SGAHSFDSSQMNMSHVVTHLSFGRMIDTRLLTDLKRLLPYLGQSHDKLDEKAFINQHEFG<br>ANVTIEHYLQIVKTEVITRRYGQEHSLTEEHEYTAHSSITQTYYLPVAKFHFELSPMQILITE<br>NPKSFSHFITNLCAIIGGVFTVAGIIDSVLHNTIRLIKKVELGKNI
BrPDI8-2	Bra035770	MVSPTKLKSMDFYRKIPRDLTEASLSGAGLSIVAALVMMLLFGMELSSYLAVNTTTAVVV<br>DKSADGDFLRIHFNISFPALSCEFASVDVSDVLGTNRLNITKTIRKFPIDPHLKTTGEEFHSG<br>HGSHDINHGEE<br>TKEEIPDGSVPLVSSSFDSFSKHFPLLIVNFNAPWCYWSNRLKPSWEKASSIIYHKYNPETD<br>GRVLLGSVDCTEEAELCKRNHIQGYPSIRIFRKGSDLKEDHGHHEHESYYGDRDTDSIVK<br>MVDELVAPIHPETHKLDLDGISNKTLKHLKKAPVTGGCRVEGYVRVKKVPGNLIISAHSG<br>AHSFDSSKMNMSHVVSHLSFGRMFSPRLLTDMRRLLPYIGQSHDKLNEKAFINQHEFGAN<br>VTIEHYLQVVKTEVITRRTAQEHSLVEEYEYTAHSSIAQTYYLPVAKFHFELSPMQIMITEN<br>PKSFSHFITNLCAIIGGVFTVAGILDSIFHNTIRLVKKVELGKNF

BrPDI8-3	Bra019071	MVSTSRIKSVDFYRKIPRDLTEATLSGAGLSIVAALSMLFLFGMELNNYLAVSTTTSIIVDRSSDGDFLRMDFNISFPSLSCEFASVDVSDVLGTNRLNVTKTIRKFSIDSNLRPTGSEFHSGEVLSHVNHDEAGEEVVEDSVSLTSRNFDTLLHQFPISVVNFYAPWCYWCNLLKPSWEKAAKQIKERYDPEMDGRVILAKVDCTQEADLCRKNHIQGYPSIRIFRQGSDLKDNAHHDHESYYGDRDTESLVKMVIGLVEPIHLEPHKLALEDKSDNASKTLKKAPSTGGCRIEGYIRVKKVPGNLMVSARSGSHSFDSTQMNMSHVVNHLSFGRKILPQTFTDLKRLSPYLGQSHDRLNGRPFINQRDLGPNVTIEHYLQIVKTEVLKSNGHAMVEEYEYTAHSSVAHSYYLPVAKFHFELSPMQVLITENSRSFSHFITNVCAIIGGVFTVAGILDSILHQTMTLMKKIELGKNF
BrPDI8-4	Bra010413	MISPRKIKSVDFYRKIPRDLTEASLSGAGLSIVAALSMLLLFGMELSSYLTVSTTTSIIIDRSSDGDFLRMDFNISFPSVSCEFASVDVSDVLGTNRLNVTKTIRKFSIDSNLRPTGSEFHSGEFLSRVNHGDESAEELVEGSVSLGARNFDTFLHQYPISVVNFYAPWCYWCNLLKPSWEKAANQIKERYDPEMDGRVILAKVDCTQEADLCRRNHIQGYPSIRIFRKGSDLRDDNAHHDHESYYGDRDTESLVKMVIGLVEPIHLEPHKLALEDKSGNASKTLKKAPSTGGCRIEGYMRVKKVPGNLMVSARSESHSFDTSQMNMSHVVNHLSFGKRILPEAFSDLKRLAPYLGGSHNRLDDRSFINQHDLGPNVTIEHYLQIVKTEVLKSNGHAMIEEYEYTAHSSVAHTYYLPVAKFHFELSAMQVLITENSKSFSHFITNVCAIIGGVFTVAGILDSILHQTMTLMKKIELGKNF
BrPDI8-5	Bra030465	MVSTTKIKSVDFYRKIPRDLTEASLSGAGLSIIAALAMVFLFGMELSTYLAVTTNTSVIVDNSSDGDFLRIDFNVSFPSLSCEFASVDVSNVLGTKRLNLTKTIKKVPIDPYLRATGAEVHSTSGLHLINHGDEDHGNNTYAAIPLTGATFDKFSHHFQILVVNFYAPWCYWSNRLKPSWEKAAEITRQRYNPETDGRVLLGSVDCTEETTLCKRNHIQGYPSIRIFRKGSDLKEDHGHHEHESYHGDRDTESILKMVEELLKPIKKEDHKLALDGKTDNVVSGIKKAPVSGGCRIVGYVRAKKVPGEIIISAHSGAHSFDASQMNMSHYVSHLTFGKMISERLLTDMKRLMPYLGLSHDRLNSKWFVNEGQFAANVTIEHYLQVVKTEVVSRRFGQEHSVIEEYEYTAHSSVAHGYYYPVAKFRFDLSPMQVLISENPKSFSHFITNVCAIIGGVFTVAGILDSIFQNTFRLVKKIELGKNI
BrPDI8-6	Bra018881	MVSTTKIKSVDFYRKIPRDLTEASLSGAGLSIIAALAMMFLFGMELSTYLAVTTQTSVVVDNSSDDDFLQIDFNVSFPALSCEFATFEVSDVLSTNRLNLTKTIKKVPIDPHLRDTGEEYHPTPDSDLINHGDEHHDDNTYAAIPLSGGTFDKISHKFPILVVNFYAPWCYWSSRLRPSWEKAAEITRQKYGPENDGRVLLGSVDCTEEPTLCTKYHIQGYPSIRIFHNGSDLRGDDGHQEHDSYHGNRDTESLVKMVEELLRPIKKFDGTTNHAASRIRKAPVSGGCRIEGYVRAKKVPGELVISAVSGSHSFDASRMNMTHFVNHLSFGRLISDRLLTDMKRLLPYLGLSHDRLNGKWFVNEGKFAANVTIEHYLQVVKTEVVSRRFGQEHSVIEEYEYTAHSSVAHGYYYPVAKFHFDLSPMQVLISENPKSFSHFITNVCAIIGGVFTVAGILDSIFQSTYGIMKKVELGKNF
BrPDI9-1	Bra026786	MSLIHLFLLVSLVSLEADATTSFSSGSRSILRDIGSNVIADHKDNAVELNATNFDSVFQDTSAKFAVLEFFAHWCPACRNYKPHYEKVARLFNGPEAVHPGTVLMTRVDCAAKMNIKLCDKFSIKRYPMLFWGPPSKFVGGSWEPKQEKSEILVVEEWRTADLLLGWINKQLGSSYGLDDQKVGNDHLLPNISDHEQISQAVFDIEEATEEAFDIILSLKAIKSSETGASFIRFLQLLVPHHPSKRCRKGSAEILMNFDDLCPAGECSYDSGVNNTLRNFHICGKDLPHGYYMFCRGSKNETRGFSCGLWILMHSLSVRIEDGESQFAFTTLCDFINNFFMCDECRRHFHDMCLSVKTPFKKARDVVLWLWSTHNKVNERLKKDEDSLGTGDPKFPKIIWPPKQLCPSCYLTSTGENIDWDHDEVYKFLKRYYGEKLVSSYKKNTGGVSKEEVVVAAAEEMSVPRNALVVPVGAALAIALASCAFGALACYWRTQQKNRKHHHNPHYLRRYSSNYLVMNTFSNIESEREKER

BrPDI9-2	Bra014330	MSLAHLVLFAGLLSLVILASSSSSSSSSPGSRSILRDISGENADQKDRAVELNSSNFDSVLSD<br>TPAKYAVVEFFAHWCPACRNYKPHYEKVARLFNGPDAIHPGIVLMTRVDCAMKTNTNLC<br>DRFSVSHYPMLLWGSPTKFVSGSGEPKKEKSEIVVIDDARTAERLLKWINKQTQSSYGLD<br>DKKFENEHVRTNITDYKQISQAVYDIEEATAEAFDIILSNKVIKSSETSASFIRFIQLLAAHH<br>ASRRCRKGAAEILVNYDDLCPS<br>GKCSYEASGGKDTLGSFPICGKDLPRGYYMFCRGSKNDTRGFSCGLWVLFHSLSVRIEDG<br>ESQFAFNTICDFVNNFFMCDECRLHFNDMCLSVKTPFKKARDFVLWVWSTHNKVNERLM<br>KDEASLGSGDPEFPKIIWPPRALCPSCYLSSDEKSIEWDHDNVYKFLKSYYGPKLVSLYKE<br>KSVVGSKEETVSATAEDLTVATNALVVPVGAALAIAVASCAFGALACYWRTQQKNRKY<br>CRGEAGTEVLEAELLMFP
BrPDI10-1	Bra001092	MDLHLPILLLCVIAASCFPSGLASSPVDSSVCNHEFELFRFDLASKCPPSLRPSPPIEVDGDS<br>LDRLMALNHDDGNAYVSVLFYASWCPFSRAVRTKFDMLSLMFPQIQHLAVEHSQALPSV<br>FSRYGIHSLPSILMVNQTLKARYHGRKDLTSLIEFYDESTGLKPVQYVAEGEPATTLDATD<br>GSLITWLRNGTSISEIFKRDPFLVLSLLFICIQVAILVFPIAESRMKALWASYAPNLNLERFG<br>EVSQVFSRALHMVDVRRLWLKLR<br>LVKTRSFHERAKNAQAWASSLASVSLGQTSSDQS
BrPDI10-2	Bra031969	MDLRVPILFFLLSTIYFPSVLASSSPVDFSVCNHEFELFRFDLDSKCPPSLHPAPPLQVDGET<br>LDRLMGLNYDANGYMSVLFYASWCPFSRAVRPKFDMLSSMFPLIQHLAVEHSQALPSVF<br>SRYGIHSLPSILI<br>VNRTSKARYHGQKDLTSLIEFYEESTGLKPVQYVAEAEPTTSLDSTDGNLITWLRKGTSIS<br>EIFRQDPFLVLSLLFICLQMAILVFPIAESRMKALWASYVSNLNLERFGEISQLFSRALHMV<br>DVRRLWLKLRLV<br>KTRNFHERAKNAQAWASSLASVSLGQASSDQS
BrPDI10-3	Bra036758	MDLRVPFLLLIAVSCFPSSFASSSPSVCNHESELFRFDIHSKCPPSMYPTPPIEIDGDSLDRLM<br>ALHHDGNAYVSVLFYASWCPFSRALRSKFDTLSSMFPQIHHLALDHSQALPSVFSRYGIHS<br>LPSILMVNQTSKARYHGRKDLTSLIEFYEESTGLKPVQYVSEPEPTTSVDATDGNMITWLR<br>KGTSISEVFKGDPFLVLSLLFVCLQAAILVFPMAEQRLKALWASYVPNLNLERFGEVSQVF<br>RRAVHMVDVRRLWLKLTLVK<br>TRSFHERAKNARAWASSLASVSLGQTSSNQS
BrPDI10-4	Bra036429	MEKGILLLVLVILFGNLMFTAVDGVSVRAPICAMRSVKDYALGFREQSCPFYGDELADRP<br>HFVVVTEGDERWLQTALDMIHKNKCDYVALLFYASWCPFSRSFTPSFDLISSLYSSIPHFAI<br>KESSVKPSTLSK<br>YGVHGFPTLLLMNSTMRARYRGTRMLDSLVAFYRDVTVSELTRKLLGIETLDKTSLEKSL<br>LVPHLGNENNTEPENCPFTWARSPENMLRQETYLTLATVFVLLRLLYFVFPALVVFAKFT<br>WPRIAQNMRLES<br>LQEHTVGFLSRLCMYLKEPCKRSNLQGGAMNARAWASKSLATVSIGESSSSNSRASSASQ
BrPDI11-1	Bra019406	MALAINVSSSSSSISTSSFPSSDLKAPQIGSLRLSDRINVSSASLSLSGKRSSVKALNVQSITK<br>ESMVPPQAASMVASEIREKVDVIEVEDFEELAKKLETASPLEIMDKALENFGNDIAIAFSG<br>AEDVALIEYAHL<br>TGRPYRVFSLDTGRLNPETYRLFDTVEKHYGIRIEYMFPDAVEVQALVRSKGLFSFYEDG<br>HQECCRIRKVRPLRRALKGLRAWITGQRKDQSPGTRSEIPVVQVDPVFEGLDGGAGSLVK<br>WNPVANVEGNDVWSFLRTMDVPVNTLHAAGYVSIGCEPCTRAVLPGQHEREGRWWWE<br>DAKAKECGLHKGNIKESSNGNNAAVNGNGTTSTVDDIFKSENVVSLSRQGIENLMKLENR<br>KEAWIVVLYAPWCPFCQAMEASFDELADKLRGGDGVKVAKFRADGDQKEFAKSELQLG<br>SFPTILVFPKNSSRPIKYPSEKRDVDSLTSFLNLVR

BrPDI11-2	Bra013579	MALAINVSSSSSSAISTSSFPSSELKAPRIGSLRLSDRVNVSTASLSLSGKRSSSVKPLNVQSI<br>AKESFVPSQAASVVASEVTEKLDVVEVEDFEELAKSLETASPLEIMDKALEKFGNDIAIAF<br>SGAEDVALIEYA<br>HLTGRPFRVFSLDTGRLNPETYRLFDTVEKHYGIRIEYMFPDAVEVQALVRNKGLFSFYED<br>GHQECCRIRKVRPLRRALKGLRAWITGQRKDQSPGTRSEIPVVQVDPVFEGLDGGAGSLV<br>KWNPVANVEGNDVWNFLRTMDVPVNTLHAAGYVSIGCEPCTRAVLPGQHEREGRWWW<br>EDAKAKECGLHKGNIKENSNGNANANVNGTSSTVADIFKSENVVSLSRQGIENLMKLENR<br>KEAWIVVLYAPWCPFCQAMEGSFDELADKLGGSGVKVAKFRADGDQKEFAKRELQLGS<br>FPTILVFPKNSSRPIKYPSEKRDVDSLTSFLNLVR
BrPDI11-3	Bra034466	MALAVTSSSTAISGSSFSRSGPCSDRKALQICSFRLSDLSHVSQRRYSLKAESPPTRNDSLV<br>TRASTLITPGVEEKEEDVEDFEQLAKKLEEASPLEIMDKALQRFGSNIAIAFSGAEDVALIE<br>YARLTGRPFRVFS<br>LDTGRLNPETYRLFDAVEKQYGIRIEYTFPDAVEVQALVRNKGLFSFYEDGHQECCRVRK<br>VRPLRRALKGLKAWITGQRKDQSPGTRSEIPIVQVDPVFEGLDGGVGSLVKWNPLANVEG<br>GDVWNFLRTMDVPVNALHAQGYVSIGCEPCTRPVLPGQHEREGRWWWEDAKAKECGL<br>HKGNIKKEDDSTTADLAPAIVHDIFESSNVVALSRGGIENLLKLGNRKEPWLVVLYAPWC<br>PFCQAMEASYVELAEKLAVKGIKVAKFRADGDQKEFAKQELQLGSFPTILLFPKSAPRAIK<br>YPSEHRDVDSLMSFVNLLRSVRKHDDKELVLELMATLTRGRESY
BrPDI11-4	Bra029505	MAMAATVSSSGITSSAFSPSVISSEPKVSQIGSLKLLDRVSLTTPMSLKKRSSVKPLNAEPK<br>RNDSMVPLAATMVAQVAEEVVETEDFAELAEKLENASPLEIMDKALEMFGNDIAIAFSGA<br>EDVALIEYAHL<br>TGRPYRVFSLDTGRLNPETYRFFDAVEKHYGIRIEYMFPDSVEVQGLVRSKGLFSFYEDGH<br>QECCRVRKVRPLRRALKGLRGWITGQRKDQSPGTRSEIPVVQVDPVFEGLDGGAGSLVK<br>WNPVANVEGSDVWSFLRTMDVPVNTLHAAGYVSIGCEPCTRAVLPGQHEREGRWWWE<br>DAKAKECGLHKGNIKENANVNGESKPVVEDIFKSENVVALSRQGIENLVKLENRKEPWIV<br>VLYAPWCPFCQAMEASYDEMADKLAGSGVKVAKFRADGEQKEFAKQELQLGSFPTILVF<br>PKNSSRPIKYPSEKRDVDSLTSFLNLVR
ZmPDIL1-1	GRMZM2G091481_T01	MAIRSKAWISLLLALAVALSARAEEEPAAAAEGEAVLTLDVDSFDEAVAKHPFMVVEFY<br>APWCGHCKKLAPEYENAAKALSKHDPPIVLAKVDANEEKNRPLATKYEIQGFPTIKIFRD<br>QGKNIQEYKGPRE<br>ADGIVDYLKKQVGPASKEIKSPEDATALIDDKKIYIVGIFAEFSGTEFTNFMEVAEKLRSDY<br>DFGHTLHANHLPRGDAAVERPLVRLLKPFDELVVDSKDFDVAALMKFIDASTIPRVVTFD<br>KNPDNHPYLM<br>KFFQSSAPKAMLFLNFSTGPFDSFKSAYSAAAEEFKDKEIKFLIGDIEASQGAFQYFGLKED<br>QTPLILIQDGDSKKFLKVHVEADQIVAWLKEYFDGKLTPFRKSEPIPEVNNEPVKVVVADN<br>VHDFVFKSGKN<br>VLIEFYAPWCGHCKKLAPILDEAATTLQSDEEVVIAKMDATANDVPSEFDVQGYPTLYFV<br>TPSGKVTSYDSGRTADDIVDFIKKSKETAGAATTTTTQAPPASEKAAAAEPVKDEL

ZmPDIL1-2	GRMZM2G163421_T01	MAIRSKAWISLLLLALAAVLSARAEEPAAAEAEAVLTLDVDSFDEAVAKHPFMVVEFYAP<br>WCGHCKNLAPEYENAAKELSKHDPPIVLAKVDANEEKNRPLATKYEIQGFPTLKIFRNQG<br>KNIQEYKGPRE<br>ADGIVDYLKKQVGPASKEIKSAEGVAAHFDDKKIYIVGIFKEFSGTEFTNFMELAEKLSSD<br>YDFGHTLHANHLPRGDASVEGPLIRLLKPFDDLVVDSKDFDVAALEKFIDASSTPRVVTFD<br>NNPDNHPYLMK<br>FFQSSAPKAMLFLNFSTGPLDSFKSVYYAAAEEFKDKEIKFLIGDIEASQGAFQYFGLKED<br>QTPLILIQDGDSKKFLKDHIEADQIVSWLKEYFDGKLTPFKKSEPIPEVNNEPVKVVVADNI<br>HDVVFKSGKNV<br>LIEFYAPWCGHCKKLAPILEEAATTLLSDEEVVIAKMDATANDVPSEFEVQGYPTMYFVT<br>PSGKVTSYDSGRTADDIVDFINKSKETASAVQATATASGKAADAAEKTEPVKDEL
ZmPDIL2-1	GRMZM2G134889_T01	MGSTTMSPPSFPVVLLLLLLATIAAAAGSNMDEEVVDDLQYLIDNSDDIPTNDPDGWPEG<br>DYDDDDLLFQDQDQDLTGHQPEIDETHVVVLAAANFSSFLASSHHVMVEFYAPWCGHC<br>QELAPDYAAAAAHLAAHHHQAHLALAKVDATEETDLAQKYDVQGFPTILFFIDGVPRGY<br>NGARTKEAIVDWINKKLGPAVQNVTSVDEAQSILTGDDKAVLAFLDTLSGAHSDELAAA<br>SRLEDSINFYQTSTPDVAKLFHIDAAAKRPSVVLLKKEEEKLTFYDGEFKASAIAGFVSAN<br>KLPLVTTLTQETSPSIFGNPIKKQILLFAVASESTKFLPIFKEAAKPFKGKLLFVFVERDSEEV<br>GEPVADYFGITGQETTVLAYTGNEDARKFFLDGE<br>VSLEAIKDFAEGFLEDKLTPFYKSEPVPESNDGDVKIVVGKNLDLIVFDETKDVLLEIYAP<br>WCGHCQSLEPTYNNLAKHLRSVDSLVVAKMDGTTNEHPRAKSDGYPTILFYPAGKKSFE<br>PITFEGERTVVDL<br>YKFIKKHASIPFKLKRQESRTESTRAEGVKSSGTNSKDEL
ZmPDIL2-2	GRMZM2G033829_T01	MGSTRTSHPSFPVLLLFLLATAAAAGSNKAEEVDDLQYLIDNSEDIPPNDPDGWPEGGGG<br>GDYDDDLLFQDQDQDLPDYEPQIDETHVVVLTAANFSSFLAATRHVMVEFYAPWCGHCR<br>ELAPEYAAAA<br>AHLAVHHNQTDLALAKADATEETDLAQRYDVQGFPTIILFIDGVPKDYNGARTKDAIVD<br>WINKKLGPAVQDVTSVHEAERILTGDDKAVIAFLDTLTGAHSDELAAASRLEDSINFYQTS<br>IPDVAKLFHIDP<br>AAKRPSIVLLKKEEEKLTFYDGKFKASAIADFVSANKLPLVTTLTQETSPSIFGNAIKKQILL<br>FAVASESSKFLSIFKEAAKPFKGKLLFVFVERDNDEVGEPVANYFGLTGQETTVLAYTGNE<br>DARKFFLDGE<br>VSLEAIKDFAEGFLEDKLTPFYKSEPVPESNDGDVKIVVGKSLDVIVLDESKDVLLEIYAP<br>WCGHCQSLEPTYNKLAKHLSGVDSLVIAKMDGTTNEHPRAKSDGYPTILFYPAGKKSFEP<br>VTFEGERTVVDM<br>YRFIKKHASIPFKLKRQESRRESIQTDGVKDEL

ZmPDIL3-1	GRMZM2G014076_T01	MRARWAVTLLLLAVLALTASAARLDLDDDDDSGVLDELLAIDEEAERGGLLDAEGAGE<br>AVRRAQSMVLALDNDNARRAVEDHAELLLLGYAPWCERSAQLMPRFAEAAAALRAMG<br>SAVAFAKLDGER<br>YPKAAAAVGVKGFPTVLLFVNGTEHAYHGLHTKDAIVTWVRKKTGVPIIRLQSKDSAEEF<br>LKKDMTFVIGLFKNFEGADHEEFVKAATTDNEVQFVETSDTSVAKVLFPGITSEEKFVGL<br>VKSEPEKFEKFD<br>GKFEEKEILRFVELNKFPLITVFTELNSGKVYSSPIELQVFTFAEAYDFEDLESMVEEIARAF<br>KTKIMFIYVDTAEENLAKPFLTLYGLESEKKPTVTAFDTSNGAKYLMEADINANNLREFCL<br>SLLDGTLPPYH<br>KSEPLPQEKGLIEKVVGRTFDSSVLESHQNVFLEVHTPWCVDCEAISKNVEKLAKHFSGSD<br>NLKFARIDASVNEHPKLKVNNYPTLFLYLAEDKSNPIKLSKKSSVKDMAKLIKEKLQIPDV<br>ETVAAPDNVK<br>DEL
ZmPDIL4-1	GRMZM2G128171_T03	MAISQISRIFLAILLLAAAFAAAPAALADGDDVVALTESTFEKEVGKDRGALVEFYAPWC<br>GHCKKLAPEYERLGASFKKAKSVLIAKVDCDEHKSLCSKYGVSGYPTIQWFPKGSLEPKK<br>YEGQRTAEALAE<br>FLNTEGGTNVKLATIPSSVVVLTPETFDSIVLDETKDVLVEFYAPWCGHCKSLAPTYEKVA<br>SVFKLDEGVVIANLDADKHRDLAEKYGVSGFPTLKFFPKGNKAGEDYDGDRDLVDFVKF<br>INEKSGTSRDTK<br>GQLTSEAGRIASLDVLAKEFLGASGDKRKEVLSSMEEEADKLSGSAARHGKVYVTIAKKI<br>LEKGNEYTEKETKRLDRILEKVGNAYLARCLMKHPLLGQLTVQI
ZmPDIL4-2	GRMZM2G159369_T01	MAFPQISRRALGLLLVIAAAAAIVSPATADEVVALTEADFEKEVGQDRGALVEFYAPWCG<br>HCKKLAPEYEKLGASFKKAKSVLIAKVDCDEHKSVCSKYGVSGYPTIQWFPKGSLEPKKY<br>EGQRSVEALAE<br>FVNSEAGTNVKIAAIPSSVVVLTSETFDSIVLDETKDVLVEFYAPWCGHCKHLAPIYEKLA<br>SVFKQDDGVVIANIDADKHTDLAEKYGVSGFPTLKFFPKGNKAGEDYDGGRDLDDFVKFI<br>NEKCGTSRDPK<br>GHLNQEAGLVPSLNPLVKEFLNAADDKRKEVLSKIEEDVAKLSGSAAKHGKIYVTAAKKI<br>IDKGSDYTKKETERLHRMLEKSISPSKADEFIVKKNILSIFSS
ZmPDIL5-1	GRMZM2G389173_T01	MRPAVVTVLLLVAAAASPAAALYSAGSPVLQLNPNNFKSKVLNSNGVVLVEFFAPWCGH<br>CKQLAPAWEKAAGVLKGVATVAALDADAHQALAQEYGIKGFPTIKVFSPGKPPVDYQG<br>ARDVKPIVEFAL<br>SQVKSLLRDRLSGKASAGSNGKTSGGSSEKSEPSASVELNSRNFDELVVKSKDLWIVEFFA<br>PWCGHCKKLAPEWKKAAKNLKGQVKLGHVDCDAEKSLMSKYKVEGFPTILVFGADKES<br>PFPYQGARVAS<br>AIESFALEQLEANSGPAEVSELTGPDVMEEKCASAAICFVSFLPDILDSKAEGRNKYLELLL<br>SVAEKFKKSPYSFVWTAAGKQANLENQVGVGGYGYPAMVALNVKKGAYAPLRSAFQR<br>DEIIEFVKEAGRG<br>GKGNLPLNDAPTVVASEPWDGKDGEVIEEDEFSLDELMGDSSSANDEL
ZmPDIL6-1	GRMZM2G073628_T01	MDLGAPARRRLPIRLLLVSLTVLVVLTARSSAEVITLTEETFSDKIKEKDTVWFVQFCVPW<br>CKHCKNLGTLWEDLGKVMEGADEIEIGQVDCGVSKPVCSKVDIHSYPTFKVFYEGEEVV<br>KYKASMDYTRTNCSVVGTLRTRTSPAATRSRTKCRSISTCFVR

ZmPDIL7-1	GRMZM2G176443_T02	MAARVLPPPPLPLVLLLLLLPLSARDTVAAGEDFPRDGRVIDLDESNFEAALGVIDFLFVD<br>FYAPWCGHCKRLAPELDEAAPMLAGLSEPIVVAKVNADKYRKLGSKYGVDGFPTLMLFI<br>HGVPIEYTGSRK<br>ADQLVRNLKKFVAPDVSILESDSAIKNFVENAGTSFPMFLGFGVNDSLIAEYGRKYKKRA<br>WFAVAKDFSEDVMVAYEFDKVPALVAIHPKYKEQSLFYGPFEENFLEDFVRQSLLPLVVP<br>INTETIKMLNDD<br>QRKVVLTILEDDSDENSTQLVKILRSAASANRDLVFGYVGIKQWDEFVETFDVSKSSQLPK<br>LLVWDRNEEYELVDGSERLEEGTDQASQISQFLEGYRAGRTTKKKISGPSFMGFLNSLVSL<br>TSLYILIFVIALL<br>FVMVYFAGQDDTPQPRRIHEE
ZmPDIL7-2	GRMZM2G007385_T01	MAMALRRLLLPLLLLVLLGLRPQSCVASGGGGGEPAEFEIPRDGSVLELDESNFEAAVRA<br>AEFLFVDFYAPWCGHCKRLAPQLDEAAAVLAGLSTPVLVAKVNADKYKKLGSKYGVDG<br>FPTLMFFDHGV<br>PSEYTGSRKADVLVENLKKLVAPDVSVLESDSSINGFVQAAGINFPLFIGFGMDESLIVEYG<br>AKYKKKAWFSTAKDFSEDVMVVYDFDKVPALVSVNPKYNEQSVFYGPFEGTFLEDFIRQ<br>SLLPATVPINRE<br>TVKLLKDDGRKVVLTILEDESDESSLQLIKVLRSAANANHDLVFGYVGVKQWEEFTETFD<br>VKVSQLPKIVVWDTKEEYEVVEGSESFIEGDYGSQVSRFLEGYREGRTTKKKVGRGSPTL<br>LGLNAVYILVL<br>LVAVLVVLMYFSAQGEEDHQPRRAHED
ZmPDIL8-1	GRMZM2G067063_T01	MISSSKLKSVDFYRKIPRDLTEASLSGAGLSIVAALAMVFLFGMELSSYLAVNTTTSVIVDR<br>SSDGEFLRIDFNMSFPALSCEFASVDVSDVLGTNRLNITKTVRKYSIDRNLVPTGSEFHPGPI<br>PILNKHGDDVEEDHVDGAFSLSSRNFDSFSHQYPVLVVNFYAPWCYWSNRLKPSWEKTA<br>KIMRERYDPEMDGRILLGKVDCTEEVELCRRNHIQGYPSIRVFRKGSDIKENQGHHDHES<br>YYGERDTESLVAAMETYVANIPKEAHALEDKSNKTVDPAKRPAPMASGCRIEGFVRVKR<br>VPGSVVISARSGHSFDPSQINVSHYVTQFSFGKRLSPRMLHEFIRLTPYLRGYHDRLAGQS<br>YTVKHGEVNANVTIEHYLQVVKTELVTQRSSKELKVLEEYEYTAHSSLVHSFYVPVVKFH<br>FEPSPMQVLVTEVPKSFSHFITNVCAIIGGVFTVAGILDSIFHNTLRMVKKIELGKNI
